# Contrasting patterns of Andean diversification among three diverse clades of Neotropical clearwing butterflies

**DOI:** 10.1002/ece3.3622

**Published:** 2018-03-25

**Authors:** Nicolas Chazot, Donna Lisa De‐Silva, Keith R. Willmott, André V. L. Freitas, Gerardo Lamas, James Mallet, Carlos E. Giraldo, Sandra Uribe, Marianne Elias

**Affiliations:** ^1^ Department of Biology Lunds Universitet Lund Sweden; ^2^ Institut de Systématique, Évolution, Biodiversité ISYEB‐UMR 7205–CNRS MNHN UPMC EPHE, Muséum national d'Histoire naturelle Sorbonne Universités Paris France; ^3^ McGuire Center for Lepidoptera and Biodiversity Florida Museum of Natural History University of Florida Gainesville FL USA; ^4^ Departamento de Biologia Animal and Museu de Zoologia Instituto de Biologia Universidade Estadual de Campinas Campinas São Paulo Brazil; ^5^ Museo de Historia Natural Universidad Nacional de San Marcos Lima Peru; ^6^ Department of Organismic and Evolutionary Biology Harvard University Cambridge MA USA; ^7^ Grupo de Investigación de Sanidad Vegetal Universidad Católica de Oriente Rionegro Colombia; ^8^ Universidad Nacional de Colombia, Sede Medellín Medellín Colombia

**Keywords:** Andes, biogeography, Dircennina, Ithomiini, Lepidoptera, Neotropics, Oleriina, trait‐dependent diversification

## Abstract

The Neotropical region is the most biodiverse on Earth, in a large part due to the highly diverse tropical Andean biota. The Andes are a potentially important driver of diversification within the mountains and for neighboring regions. We compared the role of the Andes in diversification among three subtribes of Ithomiini butterflies endemic to the Neotropics, Dircennina, Oleriina, and Godyridina. The diversification patterns of Godyridina have been studied previously. Here, we generate the first time‐calibrated phylogeny for the largest ithomiine subtribe, Dircennina, and we reanalyze a published phylogeny of Oleriina to test different biogeographic scenarios involving the Andes within an identical framework. We found common diversification patterns across the three subtribes, as well as major differences. In Dircennina and Oleriina, our results reveal a congruent pattern of diversification related to the Andes with an Andean origin, which contrasts with the Amazonian origin and multiple Andean colonizations of Godyridina. In each of the three subtribes, a clade diversified in the Northern Andes at a faster rate. Diversification within Amazonia occurred in Oleriina and Godyridina, while virtually no speciation occurred in Dircennina in this region. Dircennina was therefore characterized by higher diversification rates within the Andes compared to non‐Andean regions, while in Oleriina and Godyridina, we found no difference between these regions. Our results and discussion highlight the importance of comparative approaches in biogeographic studies.

## 1 INTRODUCTION

The formation of mountains is a major geological event that results in profound changes in the topography, climatic conditions, and water drainage that are likely to influence the timing and geography of diversification. Mountains may act as a barrier that isolates populations on both sides or forms an island‐like archipelago (e.g., Hughes & Eastwood, 2006), thereby driving vicariant speciation events. Climatic turnover and complex topography along the slopes allow the establishment of a large variety of habitats, vegetation, predator, and pathogen communities and may in turn affect diversification (Badgley, 2010). A diversity of environmental and ecological conditions provides multiple opportunities for adaptation and ecological speciation. The distribution of poikilotherm phytophagous insects for example will be directly determined by temperature, rainfall, and solar radiation (e.g., Menéndez et al., 2007), as well as by the plant community that hosts their larval stages and that is known to also act as an important driver of diversification (Ehrlich & Raven, 1964; Janz, Nylin, & Wahlberg, 2006). From a biogeographic point of view, mountain ranges not only generate local diversification along their slopes, but they can also feed adjacent areas through dispersal events, potentially enhancing diversification in neighboring regions. Assessing the timing of diversification and dispersal events with respect to mountain uplift is therefore of primary importance in understanding the origins of many modern biotas.

The formation of the Andean cordillera that extends from northern Venezuela to southern Chile has been proposed as the main driver of diversification in the Neotropical region (Hoorn et al., 2010). However, the timing and magnitude of surface uplift along the Andean cordilleras is highly controversial (see, e.g., Evenstar, Stuart, Hartley, & Tattitch, 2015 and references therein). Despite such uncertainty, it is undeniable that the formation of the Andes provided new ecological conditions along the slopes of the cordillera, modified the climate of the Neotropical region and deeply affected the formation of the Amazonian basin by depositing large quantities of sediment and modifying water drainage patterns (Hoorn et al., 2010). In many groups of birds, plants, and insects, species richness peaks along the slopes of the Andes and this region is recognized as the world's richest biodiversity hot spot (Myers, Mittermeier, Mittermeier, Da Fonseca, & Kent, 2000). The role of the Andean orogeny in generating this remarkable diversity hot spot has long been debated, with important studies in several vertebrate groups, especially birds (e.g., Alfaro, Cortés‐Ortiz, Di Fiore, & Boubli, 2015; Beckman & Witt, 2015; Brumfield & Edwards, 2007; Buckner, Alfaro, Rylands, & Alfaro, 2015; Castroviejo‐Fisher, Guayasamin, Gonzalez‐Voyer, & Vilà, 2014; Chaves, Weir, & Smith, 2011; Dantas et al., 2016; Fouquet, Santana, Haddad, Pech, & Trefaut, 2014; Hutter, Guayasamin, & Wiens, 2013; McGuire, Witt, Altshuler, & Remsen, 2007; McGuire et al., 2014; Parada, D'Elía, & Palma, 2015; Rojas, Warsi, & Dávalos, 2016; Sedano & Burns, 2010; Weir, 2006), and plants (e.g., Antonelli & Sanmartín, 2011; Givnish et al., 2014; Hughes & Eastwood, 2006; Lagomarsino, Condamine, Antonelli, Mulch, & Davis, 2016; Madriñán, Cortés, & Richardson, 2013; Moonlight et al., 2015). An improved understanding of the role of the Andes in Neotropical diversification should result from examining a large range of taxa and assessing the extent to which groups have been similarly affected or not by the Andes. In particular, insects represent the bulk of terrestrial diversity but remain under‐represented in biogeographic research, despite a number of recent studies of Neotropical butterflies (Hall, 2005; Elias et al., 2009; Casner & Pyrcz, 2010; Mullen, Savage, Wahlberg, & Willmott, 2011; Rosser, Phillimore, Huertas, Willmott, & Mallet, 2012; Condamine, Silva‐Brandão, Kergoat, & Sperling, 2012; Matos‐Maraví, Peña, Willmott, Freitas, & Wahlberg, 2013; De‐Silva, Elias, Willmott, Mallet, & Day, 2016; Chazot et al., 2016).

Several scenarios have been proposed to explain the role of the Andes in Neotropical diversification, but there has been confusion surrounding these scenarios and the actual processes underlying each of them. Chazot et al. (2016) proposed a clarified framework of four nonmutually exclusive diversification scenarios with respect to the Andean mountains, based on the assumption that a species pool of a biogeographic region results from the processes of speciation, extinction, dispersal, and the amount of time the region has been occupied. These scenarios and their predictions are as follows. (1) *Cradle scenario*. The Andes promote vicariant speciation and ecological speciation across and along the slopes, as supported for instance by the extremely high rates of speciation inferred in some Andean groups of plants (Madriñán et al. 2014). Under this scenario, Andean diversity is the result of increased speciation rates in Andean lineages compared to other regions. (2) *Museum scenario*. The Andes may have provided more stable environments during periods of climate change and hence may have saved lineages from extinction. Under such a scenario, Andean diversity is the result of lower extinction rates of Andean lineages compared to other regions (Stebbins, 1974). (3) *Species‐attractor scenario*. Lineages in areas adjacent to the Andes may have taken advantage of newly available Andean niches to colonize the slopes of the Andes multiple times (Brumfield & Edwards, 2007; Chazot et al., 2016). In this scenario, the colonization rate into the Andes is higher than the colonization rate out of the Andes. (4) *Time‐for‐speciation scenario*. If Neotropical clades historically originated in the Andes before spreading into the rest of the Neotropical region, they will have accumulated species over longer periods of time, regardless of differences in diversification and dispersal. Under such a scenario, the first colonization time of the Andes is higher than the first colonization time of non‐Andean regions (time‐for‐speciation hypothesis, Stephens & Wiens, 2003).

Here, we investigated spatial and temporal patterns of diversification and assessed support for these four biogeographic scenarios in two Neotropical butterfly clades, the ithomiine subtribes Dircennina and Oleriina (Nymphalidae: Danainae), and we compare them to published diversification patterns of a third ithomiine subtribe, Godyridina (Chazot et al., 2016). Both clades belong to the nymphalid tribe Ithomiini, one of the best‐studied groups of Neotropical butterflies (Mallarino, Bermingham, Willmott, Whinnett, & Jiggins, 2005; Brower et al., 2006; Willmott & Freitas, 2006; Elias et al., 2009; Brower, Willmott, Silva‐Brandão, Garzón‐Orduña, & Freitas, 2014; Garzón‐Orduña, Silva‐Brandão, Willmott, Freitas, & Brower, 2015; Chazot et al., 2014, 2016; De‐Silva et al., 2010, 2016, 2017). These three clades (the three largest ithomiine subtribes with 101 known species of Dircennina, 77 Godyridina, and 64 Oleriina, representing over 60% of ithomiine diversity) are endemic to the Neotropical region and occupy forest habitats from Central America to the Atlantic Forest, from the lowlands to high altitudes in the Andes. A recent study of spatial and temporal patterns of diversification in Godyridina, where nearly 60% of the species are found in the Andes, revealed that this subtribe originated probably at the interface between the upper Amazon region and the Central Andes about 17 million years ago (Chazot et al., 2016). The Godyridina diversification pattern conforms to the *species‐attractor* scenario, with repeated colonization of the Andes, and the subtribe also underwent local radiations in the Northern Andes, in the Central Andes, and in the Upper Amazon (Chazot et al., 2016). Concerning Dircennina and Oleriina, although both subtribes are diverse in Andean regions (De‐Silva et al., 2016, 2017), they show contrasting pattern of species distribution among Andean and non‐Andean regions. Dircennina are unusually species‐rich in the Andes compared to the rest of the Neotropics (63% of their diversity is in the Andes) whereas Oleriina have similar species richness in Andean and non‐Andean regions (49% of their diversity is in the Andes, with relatively high diversity also in Amazonia), which suggests the possibility of different scenarios of diversification. The three ithomiine subtribes therefore represent an excellent system for investigating how closely related clades have been affected by the Andes during their evolution and for improving our understanding of the mechanisms involved in diversification.

A phylogeny of Oleriina has been published (De‐Silva et al., 2010, 2016), and the phylogeny of the richest dircennine genus, *Pteronymia* (53 species, i.e., about half of the subtribe), has just been generated (De‐Silva et al., 2017). Here, we compile new and published sequences to confirm and revise, when needed, the taxonomy in the remaining dircennine genera, and to generate the first time‐calibrated molecular phylogeny of the entire subtribe Dircennina. For the Oleriina, we use the time‐calibrated phylogeny published by De‐Silva et al. (2016). We investigate the spatial pattern of species diversification of the two subtribes using the framework developed for the Godyridina (Chazot et al., 2016) as follows. (1) Using an explicit biogeographic model, we perform a biogeographic ancestral area reconstruction. (2) Using models of trait‐dependent diversification, we assess the support of the four biogeographic scenarios outlined above for Andean diversification: Andean lineages had a higher speciation rate (*cradle* hypothesis), Andean lineages had a lower extinction rate (*museum* hypothesis), the Andes were colonized at a higher rate (*species‐attractor* hypothesis) or earlier (*time‐for‐speciation* hypothesis) than non‐Andean regions. (3) Based on the best trait‐dependent diversification models, we infer a reconstruction of ancestral areas that is compared to that of the biogeographic model. Finally (4), we investigate diversification rates through time and across lineages using time‐dependent models of diversification. We then compare spatial and temporal patterns of diversification for the Dircennina, Oleriina, and Godyridina.

## 2 MATERIAL AND METHODS

### 2.1 Phylogenetic trees

#### 2.1.1 Sampling, genes, and PCR conditions

Our study focuses on two ithomiine subtribes, Oleriina and Dircennina. A calibrated species‐level phylogeny of Oleriina, which includes four genera: *Hyposcada*,* Oleria*,* Ollantaya,* and *Megoleria*, is available from De‐Silva et al. (2016), and this phylogeny was used in the diversification analyses. This tree includes 54 of 64 species (84% sampling) and was time‐calibrated using secondary calibrations from Wahlberg et al. (2009) (De‐Silva et al., 2016).

The subtribe Dircennina comprises 101 species (after our taxonomic revisions) forming seven genera: *Callithomia, Ceratinia, Dircenna, Episcada, Haenschia, Hyalenna,* and *Pteronymia*. In this study, we obtained DNA sequences of the mitochondrial fragment spanning the genes COI and COII (2286 bp), and nuclear genes EF1a (1254 bp), tektin (741 bp) from previous studies (Chazot et al., 2016; De‐Silva et al., 2016, 2017; Elias et al., 2009; Mallarino et al., 2005) and we additionally sequenced COI‐COII, EF1a, tektin, CAD (849 bp), GAPDH (690 bp), MDH (732 bp), and RPS2 (408 bp) (Wahlberg & Wheat, 2008) for Dircennina specimens (Appendix S1). Our final dataset comprised 170 individuals representing 86 Dircennina and 45 outgroup species. Fourteen Dircennina species were not included in our analyses (Appendix S1), because no DNA sequences could be obtained. As the taxonomy of the genus *Pteronymia* has already been revised by De‐Silva et al. (2017), here we only included one representative per species from that genus, corresponding to the consensus sequences of all individuals available for each species (see De‐Silva et al., 2017 for more details). PCR conditions followed Elias et al. (2009) for COI‐COII, EF1, and tektin and Wahlberg and Wheat (2008) for CAD, GAPDH, MDH, and RPS2.

#### 2.1.2 Dircennina individual‐level phylogeny

We aligned sequences with CodonCode Aligner v6.0.2. The molecular dataset was partitioned by gene and codon positions. We performed a maximum‐likelihood analysis including all individuals using IQ‐TREE software as implemented in the W‐ID‐TREE server (Nguyen, Schmidt, von Haeseler, & Minh, 2015; Trifinopoulos, Nguyen, von Haeseler, & Minh, 2016). IQ‐TREE automatically selected the best partition scheme, and we performed 1000 ultrafast bootstrap analyses (Minh, Nguyen, & von Haeseler, 2013). The tree can be found in Appendix S2.

#### 2.1.3 Dircennina species‐level phylogeny and dating analyses

We computed a consensus sequence for each species and gene region, resulting in a dataset containing 86 species and 44 outgroups. We used the “greedy” algorithm and linked rates implemented in PartitionFinder 1.1.1 (Lanfear, Calcott, Ho, & Guindon, 2012) to select the best models of substitution for optimized sets of nucleotides over all models implemented in BEAST. A time‐calibrated phylogeny was generated using BEAST 1.8.2 (Drummond, Suchard, Xie, & Rambaut, 2012), using the best partition scheme and uncorrelated lognormal relaxed clock for each partition. We applied eight secondary calibration points based on Nymphalidae node ages obtained from Wahlberg et al. (2009) and Solanaceae host‐plant ages following De‐Silva et al. (2017) (Appendix S3). We used conservative uniform priors for secondary calibrations, with upper and lower bounds corresponding to those of the 95% credibility intervals inferred in Wahlberg et al. (2009), or to the upper (more ancient) bound of the Solanaceae lineage age inferred by Magallón, Gómez‐Acevedo, Sánchez‐Reyes, and Hernández‐Hernández (2015) and De‐Silva et al. (2017) and zero (present), because host‐plant calibrations are maximum calibrations. Each run was performed for 100 million generations and sampled every 100,000 generations, resulting in 1,000 trees. The maximum clade credibility tree using the median of posterior distribution for node ages was extracted using TreeAnnotator (Drummond et al., 2012), applying a 10% burn‐in (Appendix S4‐S5). We also ran two independent analyses to assess the effect on node ages of using a Birth‐Death tree prior or a Yule prior. Results were extremely similar (Appendix S5), and we used the Birth‐Death tree for all analyses.

### 2.2 Biogeographic analyses

**Figure 1:**
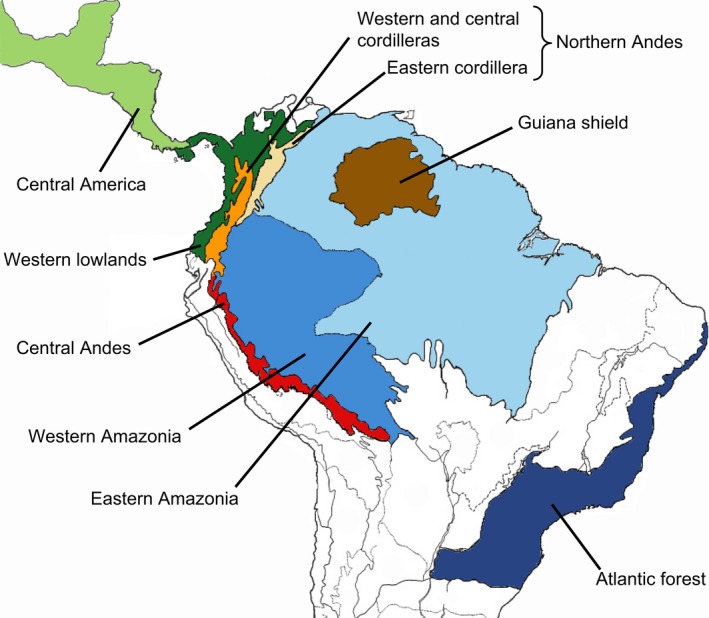
Map showing the delimitation of biogeographic areas used for biogeographic ancestral state reconstructions, modified from Morrone (2014). The western central and eastern cordilleras are referred to as the Northern Andes throughout the study

Following Chazot et al.'s (2016) analyses of Godyridina diversification, we conducted two types of biogeographic analyses for each subtribe to assess the relative support for the four scenarios of diversification (*cradle, museum, species‐attractor, time‐for‐speciation*). First, we divided the Neotropical region into nine areas to infer past distributions of ancestral lineages. We followed Morrone's (2014) classification of Neotropical biogeographic to define these nine areas (Figure 1): Central America, lowlands adjacent to the western slopes of the Northern Andes, Central Andes, western and central cordilleras of the Northern Andes, eastern cordillera of the Northern Andes, Guiana Shield, upper Amazon, lower Amazon, and the Atlantic Forest. We inferred ancestral biogeographic areas under six different models of biogeographic reconstruction. Each model accounts for different range‐changing processes, controlled by specific parameters. We used the models DIVALIKE, DEC, and BAYAREALIKE as implemented in BioGeoBEARS v.0.2.1 (Matzke, 2014), and each model was fitted with and without the founder‐event speciation parameter (j). The differences among these three models are that DIVALIKE accounts for vicariant speciation events occurring for widespread ranges, DEC accounts for sympatric events of speciation where one descendant only inherits a subset of the ancestral range and BAYAREALIKE accounts for sympatric events of speciation where the two descendants inherit the entire ancestral range. Finally, the founder‐event speciation parameter (j) accounts for speciation events where one of the descendants occupies an area that was not occupied by the ancestor. These six models for each phylogeny were compared using AIC scores. De‐Silva et al. (2016) made an ancestral biogeographic area reconstruction of Oleriina with slight differences in the delineation of the areas. To make Oleriina strictly comparable to Dircennina, we reanalyzed the biogeography according to the aforementioned procedure.

As a second step, we performed an analysis that aimed to statistically test the role of the Andes in the biogeography of the Neotropics and understand the origin of high Andean species richness. We assigned species to Andean or non‐Andean regions and fit models of character state‐dependent speciation and extinction to test whether the Andes (1) had a higher speciation rate than non‐Andean lineages, as expected under the *cradle* hypothesis, (2) had a lower extinction rate as expected under the *museum* hypothesis, (3) had a higher rate of colonization into the Andes than out of the Andes as expected under the *species‐attractor* hypothesis, or (4) were colonized before non‐Andean regions as expected under the *time‐for‐speciation* hypothesis. This follows the framework proposed by Chazot et al. (2016). Using locality and associated elevation records, species were assigned to either belonging to non‐Andean (region 1) or Andean (region 2) regions. Amazonian species whose distribution slightly overlaps with the low altitude Andean foothills were considered non‐Andean (Chazot et al., 2016). We applied the ClaSSE model of diversification, which can incorporate up to 10 parameters (Goldberg & Igic, 2012). However, we reduced the number of models following Chazot et al. (2016). We constrained to 0 the speciation parameters involving a state transition in both descending lineages (λ_122_ and λ_211_) and the anagenetic state transition rates (q_12_ and q_21_) because these parameters correspond to unrealistic biogeographic events. Therefore, the most complex model included four speciation parameters that are biogeographically meaningful (within region speciation rates: λ_111_ λ_222_, transition rates between regions: λ_121_, λ_212_) and two extinction parameters (μ_1_, μ_2_). The models fitted included all combinations of one or more parameters free to vary. The resulting 10 models were compared using the AIC scores. Differences of two units of AIC between models were considered as a significant improvement of the fit. In addition, we performed MCMC analyses on the best fitting models as a further exploration of parameter estimates (Fitzjohn 2012). We used exponential priors and a 20,000‐steps chain. Parameters converged rapidly and we discarded the first 10% steps as burn‐in before visualizing the distributions parameter estimates.

Finally, we used trait‐dependent diversification models to infer ancestral states. However, ancestral state reconstruction is not implemented in ClaSSE. Hence, we had to use the BiSSE model for ancestral state reconstruction, which only allows anagenetic state transitions. We fit the BiSSE models equivalent to those of the best fitting ClaSSE model and checked that parameter estimates were consistent with the ClaSSE analysis. Then, we used this model to make an ancestral state reconstruction that was compared to the biogeographic reconstruction.

### 2.3 Time‐dependent diversification

The ability of character state‐dependent diversification models to confidently identify correlations between diversification rates and character state has been recently challenged (Maddison & FitzJohn, 2015; Rabosky & Goldberg, 2015). In particular, Rabosky and Goldberg (2015) have pointed at the effect of diversification rate heterogeneity in the tree on the probability of detecting false positives. We performed an additional test, which aimed at shedding light on the potential effect of rate heterogeneity on the results of ClaSSE. We tested for variation of speciation/extinction rate according to major radiations having occurring in one biogeographic region. These radiations, if associated with increased diversification rate, may strongly affect the results of ClaSSE because they bear a strong phylogenetic signal. For this purpose, we chose the approach developed by Morlon, Parsons, and Plotkin (2011) to estimate diversification rates through time. This is a maximum‐likelihood method, which accommodates time‐dependent birth–death processes that can vary across a tree and the positions of the rate shifts have to be specified a priori. Then, AIC comparisons can be used to determine whether a shift significantly improves the fit of the model or not. For each subtribe, we defined a priori positions of shifts based on the time‐tree configuration and the biogeographic reconstruction. As such, we tested a single shift in Dircennina, a subclade within the genus *Pteronymia* (hereafter, *Pteronymia*‐group) that rapidly diversified during the last 5 million years, mostly in the western cordillera of the Northern Andes. For Oleriina, we investigated two potential shifts tightly linked to the biogeography of the subtribe, both within the genus *Oleria*. One shift corresponds to the subclade known as the *makrena*‐clade (De‐Silva et al., 2010, 2016; Figure 3), which diversified entirely within the western and central cordilleras of the Northern Andes. The other shift corresponds to the large Amazonian diversification of the subclade known as the *onega*‐clade (De‐Silva et al., 2010, 2016, Figure 3).

In each case, we started by fitting models on the whole tree, but refining the sampling fraction to the different subclades for which we tested diversification shifts instead of a global sampling fraction. This provided a null hypothesis to which we compared the fit of models with an increasing number of additional shifts. When we fit models on the different subclades, the stem branch was included in the subclade (as the method was originally designed, Morlon et al., 2011), but the speciation event that led to the subclade was added to the remaining background tree to keep track of this cladogenetic event. The stem branch of the background tree was not included in the analyses. For each tree (whole tree, subclades, and corresponding background trees), we fit six models: constant speciation without extinction, time‐dependent speciation without extinction, constant speciation with constant extinction, time‐dependent speciation and constant extinction, constant speciation and time‐dependent extinction, time‐dependent speciation and time‐dependent extinction, allowing to take into consideration all possible cases of constant, time‐varying or null rates. Time dependency was modeled using an exponential function. We considered the constant speciation/no extinction model as the null model. Models were compared using AIC scores. Only models with an AIC score greater than two units were considered significantly better than the null model.

## 3 RESULTS

### 3.1 Dircennina phylogenetic tree and taxonomic changes

We implemented the taxonomic changes within the genus *Pteronymia* reported in De‐Silva et al. (2017). Our individual‐level phylogenetic analyses (Appendix S2) led us to propose an additional taxonomic change in the dircennine genus *Episcada*. *Episcada striposis* Haensch, 1909 stat. rev. is now considered as a species distinct from *Episcada clausina* (Hewitson, 1876), on the basis of highly disjunct distribution (*E. striposis* occurs in the Atlantic forest of southeastern Brazil, while *E. clausina* occurs in montane forests of the tropical eastern Andes from Ecuador to Bolivia) combined with molecular divergence (Appendix S2). The two taxa were treated as conspecific by Lamas (2004) based on similar wing pattern, morphology, and allopatry. Other sympatric species of *Episcada* show weak or no morphological differences aside from wing pattern, so the lack of strong morphological differences between *E. striposis* and *E. clausina* is not unusual within the genus.

### 3.2 Historical biogeography

**Figure 2:**
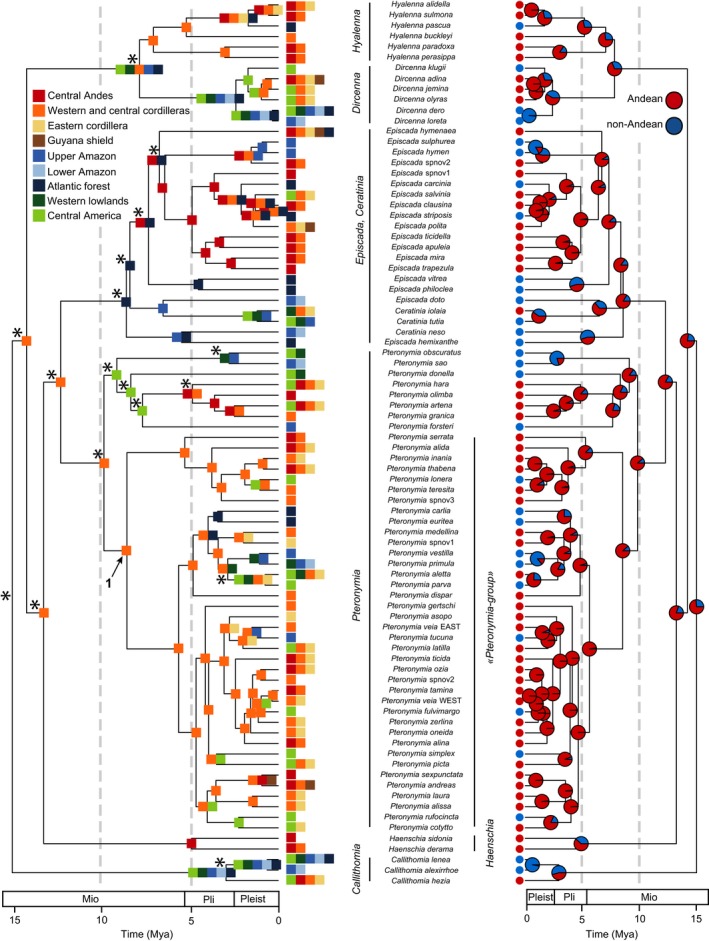
Time‐calibrated tree for Dircennina. On the left, the most likely ancestral areas inferred using the DEC model implemented in BioGeoBEARS are represented. On the right, the probabilities for each node of being Andean (red) or non‐Andean (blue) are represented. This ancestral reconstruction was obtained from the best fitting model of character state‐dependent diversification (ClaSSE analysis, see text). The numbered arrows on the left panel indicates the *Pteronymia*‐group for which we tested for a shift in diversification rate. Stars indicate unresolved ancestral state estimations. Pleist, Pleistocene; Pli, Pliocene; Mio, Miocene

We first estimated ancestral biogeographic areas using BioGeoBEARS. Among the six models fitted on Dircennina, the DEC model performed better than DIVALIKE and BAYAREALIKE and the addition of the parameter j did not improve the fit (Table 1). However, the estimation of ancestral areas under the model DEC was highly unresolved, especially at deeper nodes (Figure 2, Appendix S6). Despite this uncertainty, diversification in Dircennina mostly occurred in the western and central cordilleras of the Northern Andes, where the “*Pteronymia*‐group” radiated at least during the last 8 million years, as well as the genus *Hyalenna* (Figure 2). These two groups also showed numerous dispersal events from the Northern Andes toward the Central Andes. In the genus *Episcada*, local diversification within the Central Andes generated at least eight extant species. Central America was colonized independently at least 11 times (Figure 2) across the tree. Eleven species, all of which were included in our phylogeny, occur in the Brazilian Atlantic Forest and many result from independent colonization events with low in situ diversification. Similarly, we identified several independent dispersal events within Amazonia but very few were followed by local diversification (Figure 2).

**Table 1:** Comparison of the six models of ancestral range estimation for Dircennina and Oleriina

	Model	par	logL	AIC	∆AIC
Dircennina	DEC	2	−365.14	734.27	0
	DEC+j	3	−367.40	740.81	6.54
	BAYAREALIKE	2	−375.11	754.21	19.94
	DIVALIKE	2	−375.89	755.78	21.51
	BAYAREALIKE+j	3	−375.11	756.21	21.94
	DIVALIKE+j	3	−377.75	761.49	27.22
Oleriina	DEC+j	3	−186.76	379.51	0
	DIVALIKE+j	3	−190.73	387.45	7.94
	BAYAREALIKE	2	−196.58	397.16	17.65
	BAYAREALIKE+j	3	−196.58	399.16	19.65
	DEC	2	−198.67	401.33	21.82
	DIVALIKE	2	−200.60	405.20	25.69

par: number of parameters; logL: log‐likelihood; AIC: Akaike information criterion score; ∆AIC: difference between the model and the best fitting model.

**Figure 3:**
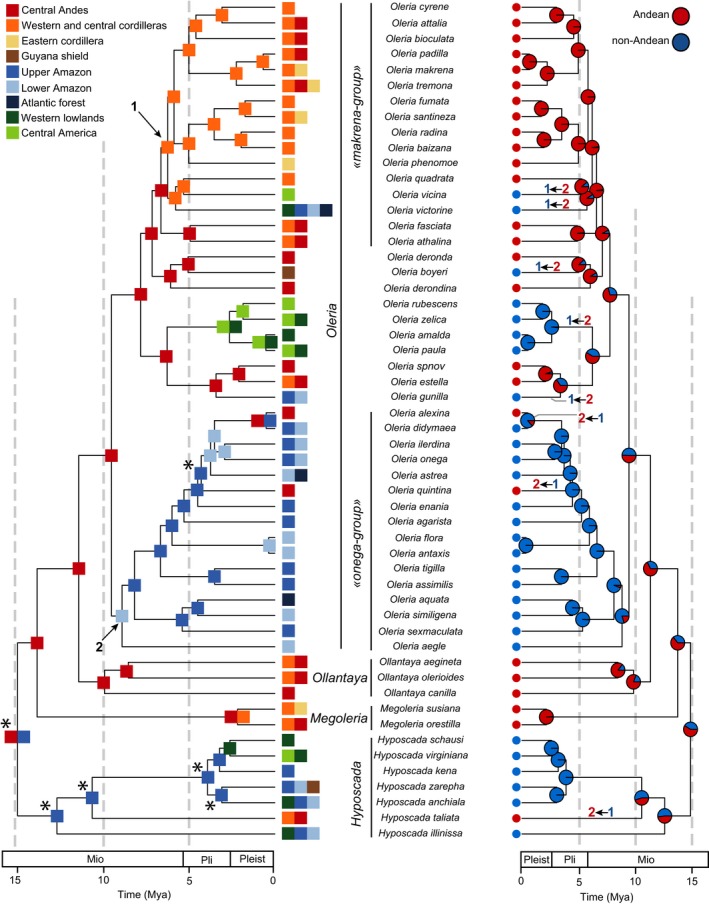
Time‐calibrated tree for Oleriina. On the left, the most likely ancestral areas inferred using the DEC model implemented in BioGeoBEARS are represented. On the right, the probabilities for each node of being Andean (red) or non‐Andean (blue) are represented. This ancestral reconstruction was obtained from the best fitting model of character state‐dependent diversification (ClaSSE analysis, see text). The numbered arrows on the left panel indicate the two subclades for which we tested for a shift in diversification rate: 1‐*makrena*‐group, 2‐*onega*‐group. On the right panel, colonization events are represented (2←1: non‐Andean toward Andean area, 1←2: Andean toward non‐Andean region). Stars indicate unresolved ancestral state estimations. Pleist, Pleistocene; Pli, Pliocene; Mio, Miocene

For Oleriina, the best model was a DEC+j (Table 1). The ancestral state estimation was better resolved than for Dircennina. Uncertainty remained at the root itself of Oleriina and for the early diversification of the genus *Hyposcada* (Figure 3, Appendix S6). This uncertainty most likely comes from the high number of regions occupied by *Hyposcada* and the broad distribution of some *Hyposcada* species. Otherwise, Oleriina appears to have diversified with a very limited number of dispersal events, leading to a very conserved biogeographic history. After the divergence of *Hyposcada*, Oleriina occupied the Central Andes before diversification split into four clearly distinct areas: Some lineages continued diversifying within the Central Andes such as the genera *Ollantaya* and *Megoleria* (Figure 3); one entire clade, the *makrena*‐group, colonized and diversified in the western cordilleras of the Northern Andes; another clade, the *onega*‐group, colonized and diversified in Amazonia; and finally the fourth clade colonized Central America leading to four extant species.

### 3.3 Trait‐dependent diversification

**Figure 4:**
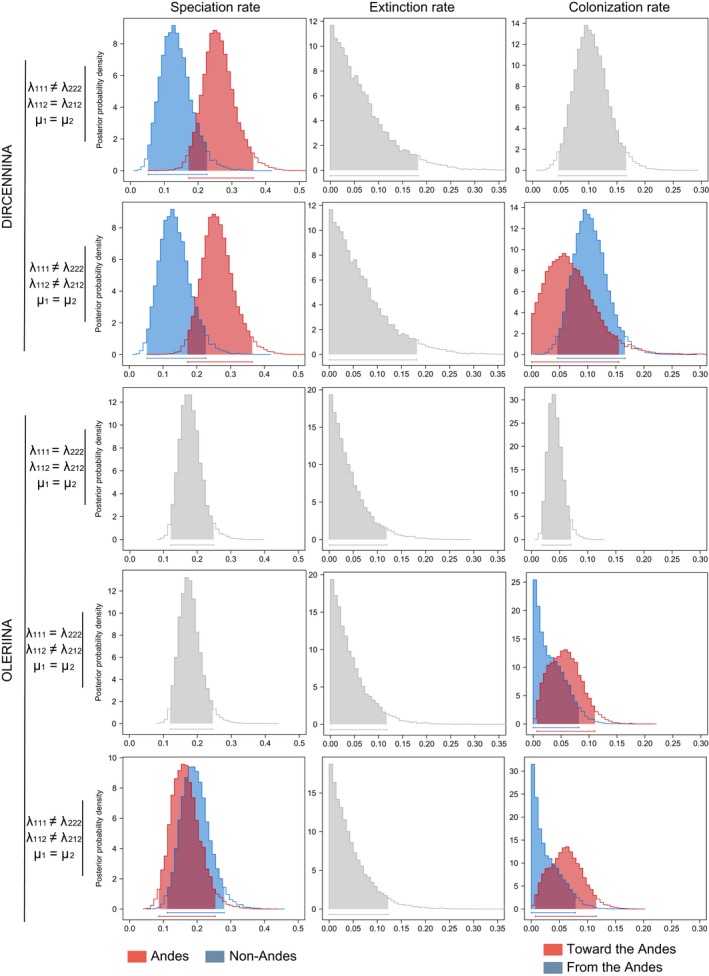
Posterior probability distribution of parameters obtained from the MCMC analyses performed on the best maximum‐likelihood fitting ClaSSE models for Dircennina and Oleriina. As two or three models had a similar explanatory power in the maximum‐likelihood analysis, we performed MCMC analyses for each model. The parameter constraints of each model are indicated on the left. First column: speciation rate, second column: extinction rate, third column: colonization rate. When the parameters were allowed to vary among regions, character state is indicated using colors: red: speciation or colonization into the Andes, blue: speciation or colonization out of the Andes

In both Dircennina and Oleriina, multiple models were within a 2‐unit AIC interval (Table 2a). For Dircennina, the best fitting model involved two different speciation rates among regions. The Andean speciation rate (λ_222_ = 0.230) was nearly twice as high as non‐Andean speciation rates (λ_111_ = 0.118). The second best fitting model recovered the same pattern but had in addition a twofold higher colonization rate from the Andes toward non‐Andean regions (λ_212_ = 0.094) than the other way round (λ_112_ = 0.047). MCMC analyses confirmed that the pattern of different speciation rates is strong (Figure 4). The distributions of colonization rate estimates overlap to a much larger extent, indicating that the signal of a twofold increase in colonization rates from the maximum‐likelihood model is much weaker (Figure 4). However, when considering the highest probability state at the nodes, we recovered 22 colonizations from the Andes toward non‐Andean regions and only one transition (poorly supported) into the Andes in the genus *Callithomia* (Figure 2). Ancestral state reconstruction of Dircennina using models of trait‐dependent diversification was highly congruent with the reconstruction inferred using BioGeoBEARS (Figure 2). Nodes inferred to be in the Andean state in ClaSSE were inferred to be in Andean areas in the BioGeoBEARS reconstruction and vice versa. The root of Dircennina was clearly inferred to be Andean, unlike in the BioGeoBEARS reconstruction.

**Table 2:** Results of ClaSSE models fitted on Dircennina (a) and Oleriina (b) sorted by increasing AIC. Constraints of each model are indicated in the first four columns

λ111/λ222		λ112/λ212	μ	*df*	logL	AIC	∆AIC	λ111	λ222	λ112	λ212	μ1	μ2
(a) Dircennina													
**λ111 ≠ λ222**	**≠**	**λ112 = λ212**	**μ1 = μ2**	**4**	−**250.061**	**508.122**	**0**	**0.118**	**0.23**	**0.079**	**0.079**	**6.10E‐06**	**6.10E‐06**
**λ111 ≠ λ222**	**≠**	**λ112 ≠ λ212**	**μ1 = μ2**	**5**	−**249.801**	**509.602**	**1.48**	**0.108**	**0.231**	**0.047**	**0.094**	**5.20E‐08**	**5.20E‐08**
λ111 ≠ λ222	≠	λ112 = λ212	μ1 ≠ μ2	5	−250.061	510.122	2	0.118	0.23	0.079	0.079	3.75E‐06	9.11E‐06
λ111 = λ222	≠	λ112 = λ212	μ1 = μ2	3	−252.359	510.718	2.596	0.187	0.187	0.077	0.077	1.00E‐09	1.00E‐09
λ111 ≠ λ222	≠	λ112 ≠ λ212	μ1 ≠ μ2	6	−249.801	511.602	3.48	0.108	0.231	0.047	0.094	9.95E‐07	2.79E‐07
λ111 = λ222	≠	λ112 = λ212	μ1 ≠ μ2	4	−251.94	511.88	3.758	0.198	0.198	0.08	0.08	0.061	1.26E‐06
λ111 = λ222	≠	λ112 ≠ λ212	μ1 = μ2	4	−252.335	512.67	4.548	0.189	0.189	0.066	0.081	5.26E‐07	5.26E‐07
λ111 = λ222	≠	λ112 ≠ λ212	μ1 ≠ μ2	5	−251.726	513.452	5.33	0.204	0.204	0.052	0.101	0.089	9.00E‐09
λ111 = λ222	=	λ112 = λ212	μ1 = μ2	2	−256.181	516.362	8.24	0.133	0.133	0.133	0.133	1.87E‐07	1.87E‐07
λ111 = λ222	=	λ112 = λ212	μ1 ≠ μ2	3	−255.657	517.314	9.192	0.108	0.108	0.047	0.094	9.95E‐07	2.79E‐07
(b) Oleriina													
**λ111 = λ222**	**≠**	**λ112** ≠ **λ212**	**μ1 = μ2**	**4**	−**169.942**	**347.884**	**0**	**0.156**	**0.156**	**0.068**	**3.48E‐07**	**7.12E‐05**	**7.12E‐05**
**λ111 = λ222**	**≠**	**λ112 = λ212**	**μ1 = μ2**	**3**	−**171.109**	**348.218**	**0.334**	**0.156**	**0.156**	**0.037**	**0.037**	**5.00E‐09**	**5.00E‐09**
**λ111** ≠ **λ222**	**≠**	**λ112** ≠ **λ212**	**μ1 = μ2**	**5**	−**169.399**	**348.798**	**0.914**	**0.177**	**0.129**	**0.07**	**1.71E‐07**	**2.96E‐07**	**2.96E‐07**
**λ111 = λ222**	**≠**	**λ112** ≠ **λ212**	**μ1** ≠ **μ2**	**5**	−**169.94**	**349.88**	**1.996**	**0.156**	**0.156**	**0.068**	**1.63E‐07**	**3.95E‐06**	**7.70E‐08**
λ111 ≠ λ222	≠	λ112 = λ212	μ1 = μ2	4	−171.075	350.15	2.266	0.163	0.15	0.037	0.037	1.90E‐07	1.90E‐07
λ111 = λ222	≠	λ112 = λ212	μ1 ≠ μ2	4	−171.109	350.218	2.334	0.156	0.156	0.037	0.037	7.89E‐06	3.14E‐06
λ111 ≠ λ222	≠	λ112 ≠ λ212	μ1 ≠ μ2	6	−169.399	350.798	2.914	0.177	0.129	0.07	4.03E‐07	1.43E‐06	5.06E‐06
λ111 ≠ λ222	≠	λ112 = λ212	μ1 ≠ μ2	5	−171.075	352.15	4.266	0.163	0.15	0.037	0.037	4.11E‐07	2.76E‐05
λ111 = λ222	=	λ112 = λ212	μ1 = μ2	2	−179.29	362.58	14.696	0.097	0.097	0.097	0.097	4.00E‐09	4.00E‐09
λ111 = λ222	=	λ112 = λ212	μ1 ≠ μ2	3	−179.291	364.582	16.698	0.177	0.177	0.07	4.00E‐06	1.43E‐06	5.06E‐06

1: non‐Andean; 2: Andean; λ111/λ222: within region speciation rates; λ112/λ212: cladogenetic transition rates; μ: extinction rates; *df*: degree of freedom (number of parameters); logL: log‐likelihood; AIC: Akaike information criterion score; ∆AIC: difference between the model and the best fitting model.

Bold values indicate the model with the lowest AIC score and all the models falling into 2‐unit AIC interval.

In Oleriina, the model with the lowest AIC had different colonization rates but three other models were found within an AIC interval of 2, involving either different speciation rates, different colonization rates or extinction rates (Table 2b). Among these four best models was also the “null” model, in which speciation rates within regions are equal, colonization rates between regions are equal, and speciation rates are different from colonization rates. We performed MCMC analyses for the first three “best” models (Figure 4). In all cases, the results of the MCMC confirmed that differences between parameters were very small with posterior distributions largely overlapping (Figure 4) contrasting with the strong difference in speciation rates found in Dircennina. In addition, ancestral state reconstructions performed using the parameters estimated by ClaSSE greatly diverged from the ancestral state reconstruction obtained with BiogeoBEARS (see also the discussion about BiSSE ancestral state reconstruction in Appendix S7). Only the ancestral state reconstruction performed with the “null” model was congruent among BiSSE, ClaSSE, and BiogeoBEARS (Appendix S7). Therefore, this model, which had the lowest number of parameters, was chosen for the ancestral state reconstruction in Figure 3 and discussed in the results. Given the results from the maximum‐likelihood analyses, the MCMC, and the ancestral state reconstructions, we conclude that neither the *cradle* hypothesis, nor the *species‐attractor* hypothesis nor the *museum* hypothesis is clearly supported in Oleriina and we discuss this result below.

### 3.4 Time‐dependent diversification

**Figure 5:**
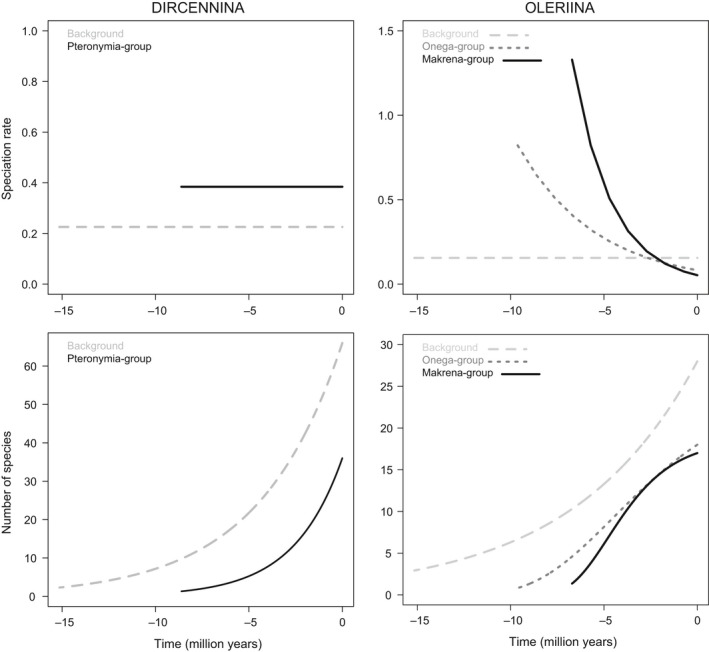
Speciation rates and diversity trajectories inferred from the best fitting models of diversification for each different subclades and background trees for Dircennina (left) and Oleriina (right). Diversity trajectories represent the number of species through time reconstructed using the best fitting model

For Dircennina, the diversification rate shift in the *Pteronymia*‐group (subclade of the genus *Pteronymia*) was significant (∆_AIC_ = 3.6 compared with null model, Table 3a and Appendix S8). For this subclade, the lowest AIC corresponded to a model of time‐dependent speciation rate, but it was not significantly different from the null model (constant speciation without extinction, Appendix S8). The speciation rate inferred by the constant speciation rate model was 0.384 (Figure 5). In the background, a model of time‐dependent speciation and extinction had the lowest AIC score, but it was not significantly different from the null model (Table 3a and Appendix S8). The speciation rate estimated for the background process by the constant speciation rate model was 0.225, that is, lower than that of the *Pteronymia*‐group (Figure 5). Consequently, diversity trajectories reconstructed from these models show species rapidly accumulating during the last 5 million years before present because of the constant speciation rates through time (Figure 5).

**Table 3:** Results of time‐dependent models of diversification fitted on the different partitions: 0 shift, 1 shift, 2 shifts. For each subclade or background tree, only the best fitting model is shown (see [Link], [Link], [Link], [Link], [Link], [Link])

(a) Dircennina									
		Model	par	logL	AIC	λ	par	Joint logL	Joint AIC
0 shift	Whole tree	BCST	1	−198.53	399.07	0.264	1	−198.53	399.07
1 shift	Background	BCST	1	−134.85	271.70	0.225	2	−195.72	**395.45**
	*Pteronymia*‐group	BCST	1	−60.87	123.74	0.384			

(b) Oleriina										
		Model	par	logL	AIC	λ	α	par	Joint logL	Joint AIC
0 shift	Whole tree	BCST	1	−140.43	282.87	0.188		1	−140.43	282.87
1 shift	Background	BCST	1	−100.71	203.42	0.181		3	−137.63	281.27
	*onega*‐group	BVAR	2	−36.92	77.84	0.082	0.239			
1 shift	Background	BCST	1	−107.52	217.04	0.172		3	−135.20	276.39
	*makrena*‐group	BVAR	2	−27.68	59.35	0.053	0.481			
2 shifts	Background	BCST	1	−67.51	137.02	0.155		5	−132.11	**274.22**
	*onega*‐group	BVAR	2	−36.92	77.84	0.082	0.239			
	*makrena*‐group	BVAR	2	−27.68	59.35	0.053	0.481			

BCST: constant speciation; BVAR: time‐dependent speciation; DCST: constant extinction; DVAR: time‐dependent extinction; λ: speciation rate at present; α: coefficient of time variation of the speciation rate; μ: extinction rate at present; ß: coefficient of time variation of the extinction rate.

For Oleriina, a diversification rate shift for the *makrena*‐group improved the fit compared to the null model (∆_AIC_ = 6.47 with null model, Table 3b and AppendixS8). The addition of another shift for the *onega*‐group also significantly improved the model, although the difference was not as important as for the *makrena‐*group (∆_AIC_ = 1.6 with the null model). The best model was obtained when both shifts were incorporated (∆_AIC_ = 8.65 with the null model, ∆_AIC_ = 2.17 with the model with only a shift for the *makrena‐*group, Figure 5). For the *makrena‐*group, the diversification model with the lowest AIC had a time‐dependent speciation rate with no extinction and it was significantly better than the null model (∆_AIC_ = 6.86 with the null model, Table 3b and Appendix S8). This model shows an initial speciation rate of 1.337, which rapidly decreases to 0.053 at present (Figure 5). The best model for the *onega*‐group was also a time‐dependent speciation rate with no extinction, with an initial rate of 0.821 at the root of the clade followed by a decrease toward 0.082 at present (Figure 5). The remaining background tree was characterized by a constant speciation rate of 0.155 without extinction, which means that both the *makrena‐*group and the *onega‐*group initially shifted toward much higher speciation rates (Table 3b, Figure 5 and Appendix S8). The accumulation of species reconstructed from the best fitting models showed that the *makrena‐*group and the *onega‐*group had a relatively similar accumulation of species during the last 6.7 million years and 9.6 million years, respectively, with a fast initial phase followed by a slow‐down during the last 1–2 million years (Figure 5).

## 4 DISCUSSION

Our current and previously published (Chazot et al., 2016) results indicate both similarities and important differences in the diversification of three closely related clades of Neotropical butterflies, the ithomiine subtribes Dircennina, Oleriina, and Godyridina (Table 4). First, we discuss in detail the pattern of diversification of Dircennina, as this is the first study on this clade. Second, as a detailed discussion of the historical biogeography of Oleriina can be found in De‐Silva et al. (2016), and the diversification patterns of the Godyridina have recently been investigated using the same framework as here (Chazot et al., 2016), we highlight the similarities and differences between the diversification patterns of the three subtribes (Table 4).

**Table 4:** Comparison of diversification patterns of Dircennina, Oleriina, and Godyridina

Biogeographic feature	Dircennina	Oleriina	Godyridina
Andean species richness/total richness	64/101	31/64	48/77
Species ranges	Broader; higher dispersal rates	Narrower; lower dispersal rates	Narrower; lower dispersal rates
Cradle hypothesis (speciation rate)	Andean>non‐AndeanStrong support	Andean<non‐AndeanNo support	Andean>non‐AndeanWeak support
Museum hypothesis (extinction rate)	No extinction	No extinction	No extinction
Time‐for‐speciation (first colonization time)	ClaSSE: AndeanBioGeoBEARS: uncertain	ClaSSE: AndeanBioGeoBEARS: both	ClaSSE: non‐AndeanBioGeoBEARS: both
Species‐attractor (colonization rate)	No support	Moderate support	Strong support
North Andean radiation?	Yes	Yes	Yes
Amazonian radiation?	No	Yes	Yes
Colonization of Central America	At least 11 independent colonizations	Only 3 independent colonizations	7 independent colonizations
Atlantic Forest colonization	At least 5 independent colonizations	Only 3 independent colonizations	9 independent colonizations

### 4.1 Diversification of Dircennina

Dircennina shows a strong asymmetrical spatial distribution of current species diversity, with a large fraction of species occurring in the Andes, mostly the northern Andes. In agreement with this pattern, we found that the diversification history of Dircennina is tightly associated with the Andes. Ancestral state estimation for deep nodes from BioGeoBEARS was unhelpful for deciphering the early history of Dircennina. However, the ancestral state estimation based on the character state speciation and extinction model clearly infers an Andean origin. Dircennina have likely occupied the Andean slopes and diversified in this area since their origin, about 15 (95% HPD: 13.39:17.09) million years ago, thereby supporting the *time‐for‐speciation* hypothesis (Figure 2). This pattern will need further investigation within a wider phylogenetic framework to infer more reliably the history of the earlier lineages.

A pattern of strong Andean diversification of Dircennina was supported by the trait‐dependent diversification analysis. Speciation rate was at least twice as high in the Andes as in non‐Andean regions, which supports the *cradle* hypothesis. Species richness of Dircennina is higher in the Northern Andes and in the Central Andes. We did not find any significant evidence of declining diversification through time, but we estimated a higher speciation rate for the *Pteronymia*‐group, which largely diversified within the Northern Andes (Figures 2 and 5). This shift may explain in a large part the difference in speciation rate between Andean and non‐Andean regions identified by ClaSSE, but also suggests a difference in diversification rates between the Central and Northern Andes. Extinction rates in both Andean or non‐Andean regions were always estimated to 0. This suggests that extinction explains very little of the current pattern of diversity and the *museum* hypothesis is not supported by our results.

Colonization events were strongly biased from Andean toward non‐Andean regions. The ancestral state reconstructions based on trait‐dependent diversification models recovered 22 out‐of‐the‐Andes dispersal events but only 1 into‐the‐Andes colonization event and the second best ClaSSE model inferred a higher rate of colonizations toward non‐Andean regions than conversely (although this was weakly supported by the MCMC analysis, Figures 2 and 4). This pattern is consistent with an *out‐of‐the‐Andes* scenario, whereby Andean lineages feed adjacent areas (not only Amazonia) through dispersal events. Such a pattern has been observed in other ithomiine clades, such as the genera *Ithomia* and *Napeogenes* (Elias et al., 2009). In the case of Dircennina, these colonization events, as well as range expansions, reached far into Central America. This is likely due to these butterflies' ability to follow mountain chains in Central America, which allowed Andean lineages adapted to mid‐ and high altitudes to colonize this region (De‐Silva et al., 2017). Conversely, colonizations of lowland regions such as the Amazon basin probably required a larger number of adaptations due to changes in climatic conditions (temperatures, moisture), host‐plants, or predators, which may have hindered colonization of such regions. In support to this hypothesis, Chazot et al. (2014) showed that the altitudinal niche of ithomiine butterflies tends to be phylogenetically conserved.

The timing of the emergence of a connection between North and South America and the timing of biotic interchanges are highly controversial (see, e.g., Bacon et al., 2015a,b; Lessios, 2008). For some time, the dominant hypothesis was a very recent emergence of land masses above sea level and the closure of the Isthmus of Panama around 4–3 million years ago (Coates & Stallard, 2013; Coates et al., 1992). However, recent publications, including fossil evidence (Bacon et al., 2015a; Bloch et al., 2016), are challenging this hypothesis and instead support a scenario in which a connection between North and South America emerged at least during the early Miocene (Farris et al., 2011; Montes et al., 2012, 2015). Our biogeographic estimation inferred one colonization that could potentially have happened as early as 9 million years ago but this was very poorly supported. All other dispersal events toward Central America occurred during the last 4 million years.

Dircennina is relatively species‐rich in the Atlantic Forest region compared to other ithomiine subtribes, but this diversity probably did not originate from local speciation events, except in a very limited number of cases. Similar to Central America, BioGeoBEARS inferred an early colonization of Atlantic Forest at the root of *Episcada *+ *Ceratinia*, but this is very poorly supported (and rejected by the BiSSE reconstruction). Most Atlantic Forest species are the result of independent colonization events (many of them from Andean ancestral lineages). This pattern appears to be relatively common, at least in butterflies, as exemplified by the ithomiine subtribe Godyridina (Chazot et al., 2016), the ithomiine genera *Ithomia* and *Napeogenes* (Elias et al., 2009), the nymphalid genera *Morpho* (Satyrinae: Morphini, Blandin & Purser, 2013), and *Taygetis* (Satyrinae: Satyrini, Matos‐Maraví et al., 2013). However, this is not true for the acraeine genus *Actinote* (Nymphalidae: Heliconiinae, Silva‐Brandão et al. 2008), which originated in the Atlantic Forest and repeatedly colonized the Amazon, the Andes and Central America.

### 4.2 Comparing diversification patterns among Dircennina, Oleriina, and Godyridina

The comparison of the three subtribes revealed both common features and differences in their patterns of diversification (Table 4). Although the origin itself of Oleriina is not clear in our biogeographic reconstruction, there is some support for an Andean origin of Oleriina (De‐Silva et al., 2016 and our trait‐dependent ancestral state reconstruction using the null model). Thus, both Dircennina and Oleriina may have spent a longer time in the Andes, supporting the *time‐for‐speciation* hypothesis. For the three subtribes, much of the recent Andean diversification occurred in the Northern Andes. Indeed, in each subtribe, we found support for increasing speciation rates in a group that diversified almost entirely in the Northern Andes (the *Pteronymia*‐group in Dircennina, the *makrena*‐group in Oleriina, and the genus *Hypomenitis* in Godyridina, Chazot et al., 2016). These three Northern Andean clades harboring increased diversification all originated between 7 and 5 million years ago. These examples point at a major difference between the faunas of Central and Northern Andes. Central Andean diversity appears to be old, having accumulated species at a relatively low pace. Northern Andean diversity appears to be younger and to have resulted from rapid bursts of local diversification. The Northern Andes is fairly young compared to the Central Andes (Hoorn et al., 2010). They also show a relatively high complexity with the Ithomiini distributed along three parallel cordilleras, which might have promoted some allopatric divergence and ecological differentiation, whereas in the Central Andes, Ithomiini only occur along the Eastern cordillera (climatic conditions in the Central and Western cordilleras are not suitable for these butterflies). Other ecological characteristics may differ in the Northern Andes from the Central Andes, such as different host‐plant and predator communities. New biotic interactions in the Northern Andes may have driven adaptive diversification in this region. We cannot rule out that the pattern of increased speciation in the Andes identified with ClaSSE in Dircennina, and to a lesser extent, Godyridina is actually mostly driven by independent Northern Andean radiations (Table 4). We can identify only few clear allopatric speciation events driven by the Andes. For lowland lineages, in Oleriina, *Hyposcada schausi* and *Hyposcada virginiana* are trans‐Andean lineages that diverged from the Amazonian *Hyposcada kena* ca. 3.2 million years ago, *that is,* the latest period of uplift in the Northern Andes. In Dircennina, trans‐Andean *Pteronymia obscuratus* also diverged recently from the Amazonian *Pteronymia sao*, ca. 2.9 million years ago, and trans‐Andean *Pteronymia latilla* diverged from the cis‐Andean *Pteronymia veia* “eastern lineage” and *Pteronymia tucuna* ca. 2.0 million years ago.

Comparing the patterns of diversification of Dircennina, Oleriina, and Godyridina also reveals important differences (Table 4). Diversification of Oleriina is undoubtedly much less tightly associated with the Andean region than that of Dircennina and, to a lesser extent, Godyridina. About half of the species of Oleriina currently occur in non‐Andean regions. The hypothesis of the Andes acting as a cradle (i.e., increasing Andean speciation rate) is clearly not supported for Oleriina, in sharp contrast with the Dircennina; while in Godyridina, Chazot et al. (2016) found a weak support for the cradle hypothesis. In Oleriina, we found an increasing speciation rate in the north Andean *makrena*‐group, but Oleriina and Godyridina differ from Dircennina by showing at least one Amazonian lowland radiation that was characterized by a burst of diversification (*onega*‐group in Oleriina, *Brevioleria* clade in Godyridina). These events, accompanied by a higher speciation rate compared to that of the background, show that regions in non‐Andean areas have also promoted fast speciation, which tempers the Andean *cradle* hypothesis as an explanation for overall high Neotropical biodiversity. In addition, most species of Oleriina have narrow geographic ranges compared to most Dircennina species, with a large proportion of species distributed in only one or two of the biogeographic areas defined here. This suggests lower migration rates or migration distances, promoting isolation‐by‐distance and therefore local diversification (De‐Silva et al., 2016), and may explain the low number of dispersal events observed in this subtribe compared to Dircennina and Godyridina.

Among the three Ithomiini subtribes, only Godyridina supports the s*pecies‐attractor* hypothesis. Chazot et al. (2016) showed that a higher number of colonizations into the Andes had most likely contributed to the current high Andean diversity in that group. Instead, both Dircennina (22 out‐of‐the‐Andes dispersal events, 3 into‐of‐the‐Andes dispersal event, Figure 2) and to a much lesser extent Oleriina (5 out‐of‐the‐Andes dispersal events, 3 into‐the‐Andes dispersal events, Figure 3) reveal a pattern of Andean diversity contributing to that of adjacent areas. Dircennina, Oleriina, and Godyridina (Chazot et al., 2016) may have all originated in the Andes ~17–15 million years ago. During most of the early and mid‐Miocene, the upper Amazon region was covered by the large Pebas wetland (Hoorn et al., 2010; Wesselingh & Salo, 2006). This region, which may have even been connected to the Pacific through the Western Andean Portal (a low altitudinal gap between the Central and Northern Andes, Antonelli, Nylander, Persson, & Sanmartín, 2009) and to the Caribbean Sea (Wesselingh & Salo, 2006), likely received episodic marine incursions. As previously suggested (e.g., Chazot et al., 2016; Hughes et al. 2013, Wesselingh & Salo, 2006), this particular ecosystem, which was drained during the late Miocene (~10–8 million years ago), may have significantly influenced the timing of interchanges between the Andean region and the Amazonian basin by preventing lineages dispersing between Central and Northern Andes and between the Andes and the Amazonian basin. In addition, this system may have also restricted upper Amazonian diversification to the edges of the Pebas System. The timing of the first colonization of the Northern Andes in Dircennina, Oleriina, and Godyridina clearly follows the demise of the Western Andean Portal (Antonelli et al., 2009; Chazot et al., 2016), while colonization into and diversification within the Amazonian basin consistently coincide with the drainage of the Pebas system. Although the Dircennina, Oleriina, and Godyridina are slightly too young to allow us to ideally assess the effect of the Pebas on restricting dispersal or driving extinctions (little diversification occurred before the demise of the Pebas system), our results support an association between dispersal/diversification and the demise of the Pebas.

### 4.3 Comparison beyond the Ithomiini

A large number of publications have now addressed the question of the role of the Andes in the biogeography of the Neotropical region. The *cradle* hypothesis has been strongly supported in some groups of plants, and several studies have revealed extremely high speciation rates, such as in the Andean genus *Lupinus* (Fabaceae) (Hughes & Eastwood, 2006) and the bellflowers (Campanulaceae) (Lagomarsino et al., 2016). The high Andean Páramo ecosystem (2800–4700 m) has been reported to host the fastest speciation rates among Earth's biodiversity hot spots (Madriñán et al. 2014). However, this later study cannot be directly compared to our results supporting the *cradle* hypothesis in Dircennina and to a lesser extent in Godyridina (Chazot et al., 2016). The fast speciation rates reported in plants mostly occur in the Páramo, a geographically restricted ecosystem of high altitude in which ithomiine butterflies do not occur and which might be highly sensitive to recent Pleistocene climatic fluctuations and the timing of Andean uplift. Although the Andes affect speciation rates in many kinds of organisms, the patterns of diversification differ among lineages. In groups other than plants, the *cradle* hypothesis has found mixed support. For example, Hutter et al. (2013) did not find higher speciation rates in Andean glassfrog lineages (Centrolenidae), while Beckman and Witt (2015) found higher speciation rates in Andean goldfinches and siskins (Fringillidae). In *Heliconius* butterflies, Rosser et al. (2012) found that species richness peaked in the eastern slopes of the Andes and was characterized by very “young” species and they interpreted this pattern as a support for the *cradle* hypothesis. We did not find support for any pattern driven by extinction in the three ithomiine subtribes, and in fact, we found no signal of extinction, which argues against the *museum* hypothesis. The role of extinction has been poorly addressed perhaps because it has been rarely proposed as an explicit biogeographic scenario, but probably also because of the controversy surrounding the ability of current methods to reliably estimate extinction from molecular phylogenies of extant species (e.g., Rabosky, 2010; but see Morlon et al., 2011); hence examples are rare. For example, Hutter et al. (2013) did not find support for different extinction rates among Andean versus non‐Andean regions in glassfrogs, but Antonelli and Sanmartín (2011) reported a lower extinction rate (combined with higher speciation rate) in the species‐rich Andean subgenus *Tafalla* compared to the remaining non‐Andean Chloranthaceae. Despite being rarely proposed as an explicit scenario of Andean diversification, the *species‐attractor* hypothesis has been relatively well supported. For example, Hall (2005) found repeated speciation events across altitudes as well as colonization events into the Andes in *Ithomiola* butterflies (Riodinidae). In plants, a large number of independent colonization events of the Andean slopes have been reported, for example, in *Begonia* (Begoniaceae) (Moonlight et al., 2015) and Bromeliaceae (Givnish et al., 2014). Finally, the *time‐for‐speciation* hypothesis, which is supported by our analyses on Dircennina and Oleriina, has also been supported in other groups such as the glassfrogs (Hutter et al., 2013).

In conclusion, we show that a strict evaluation of biogeographic scenarios within a common framework allows a “comparative” biogeographic approach. This approach helps to clearly decipher which processes are shared across different groups as well as why some groups differ from others. Here, we show that, at least for Oleriina and Godyridina, the Central Andean fauna appears to be old with slow diversification rates compared to the Northern Andean fauna, which is more recent and is diversifying at a faster rate. The three subtribes also show major dispersal and diversification events associated with the demise of the Pebas system, with a remarkable convergence in the timing of events. However, major differences also appear between these groups, especially when considering Amazonian diversification. Notably, repeated independent events of rapid diversification in Amazonia question the hypothesis that the Andes acted as a cradle (i.e., driving higher speciation rate) at the global scale and instead call for further investigation on the ecological or genetic characteristics explaining why some groups radiated in Amazonia and others not.

## CONFLICT OF INTEREST

None declared.

## AUTHOR CONTRIBUTIONS

NC, ME, and DLDS conceived the study, with contributions from KRW, AVLF, and GL. KRW, AVLF, JM, CEG, SU, ME provided specimens and sequences. NC, ME, and DLDS performed the laboratory work and the analyses. NC, ME, and DLDS wrote the article with contributions from all co‐authors.

## DATA ACCESSIBILITY

All sequences used in this study will be available on GenBank, and accession codes can be found in Appendix S1.
