# Supplementary figures and images for: Contrasting patterns of Andean diversification among three diverse clades of Neotropical clearwing butterflies
Source: Ecol Evol. 2018 Mar 25;8(8):3965–82. doi: 10.1002/ece3.3622 (PMC5916281; doi:10.1002/ece3.3622)

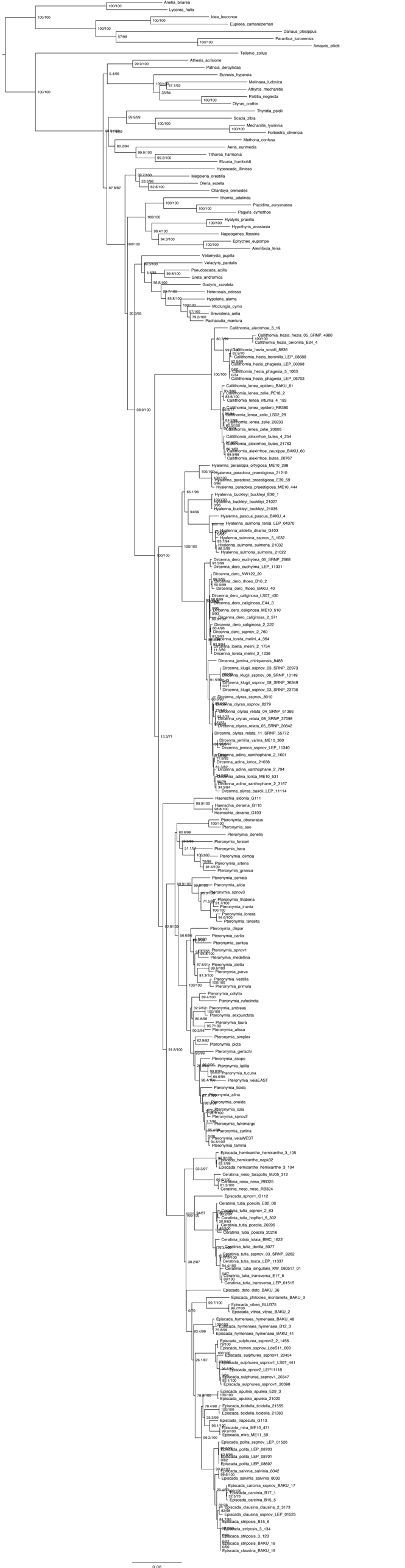

Supplement: Supplementary file 2 [file ECE3-8-3965-s002.pdf]

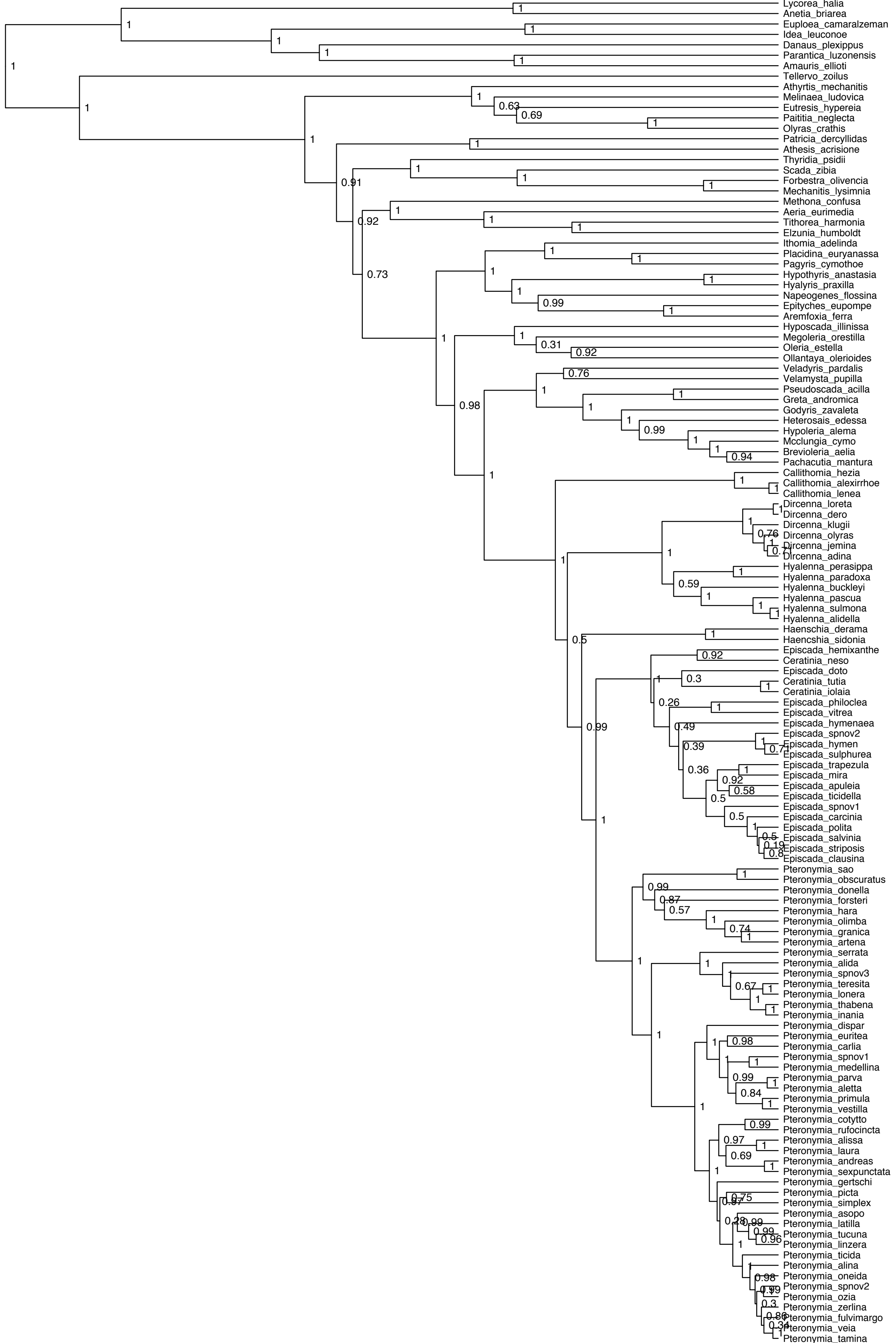

Supplement: Supplementary file 3 [file ECE3-8-3965-s003.pdf]

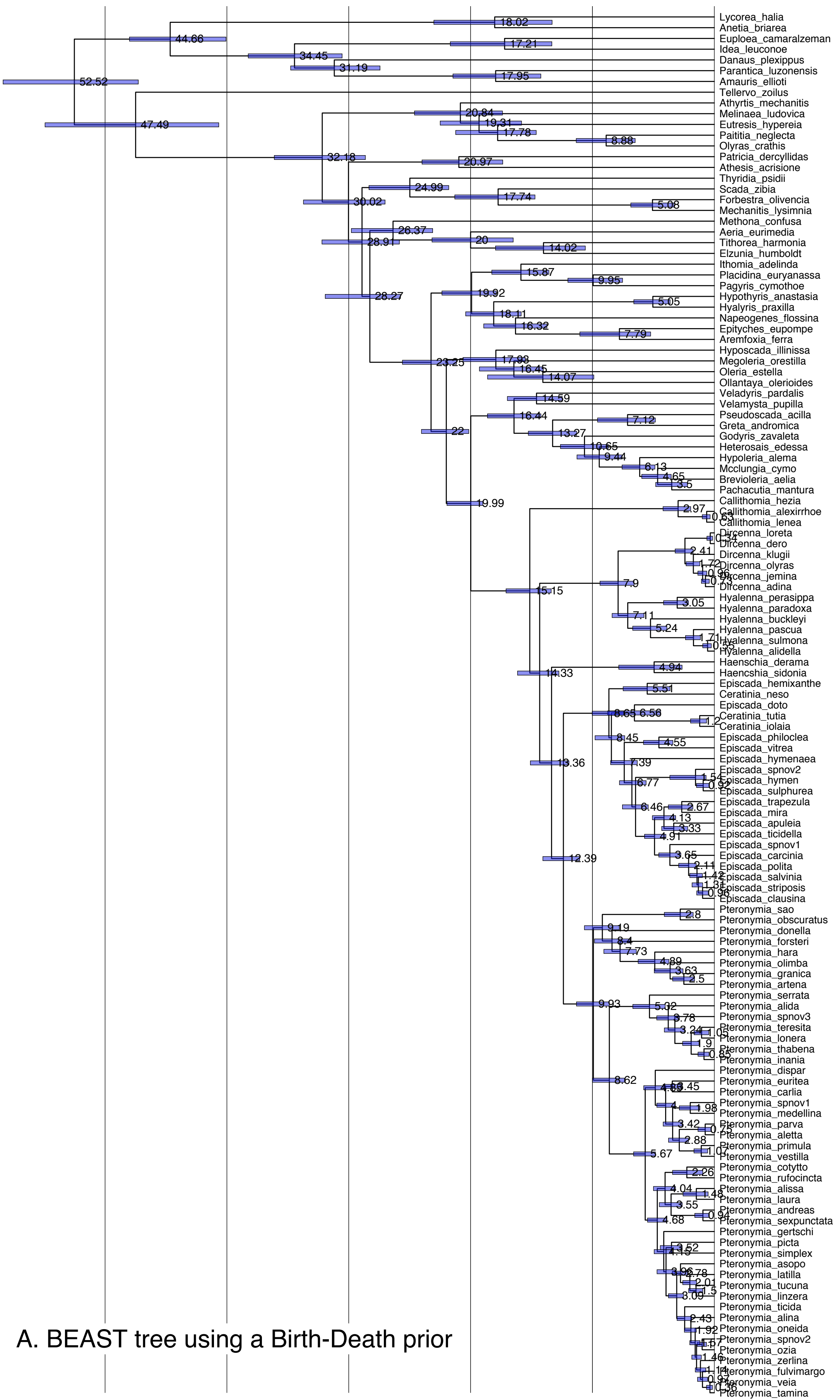

A. BEAST tree using a Birth-Death prior

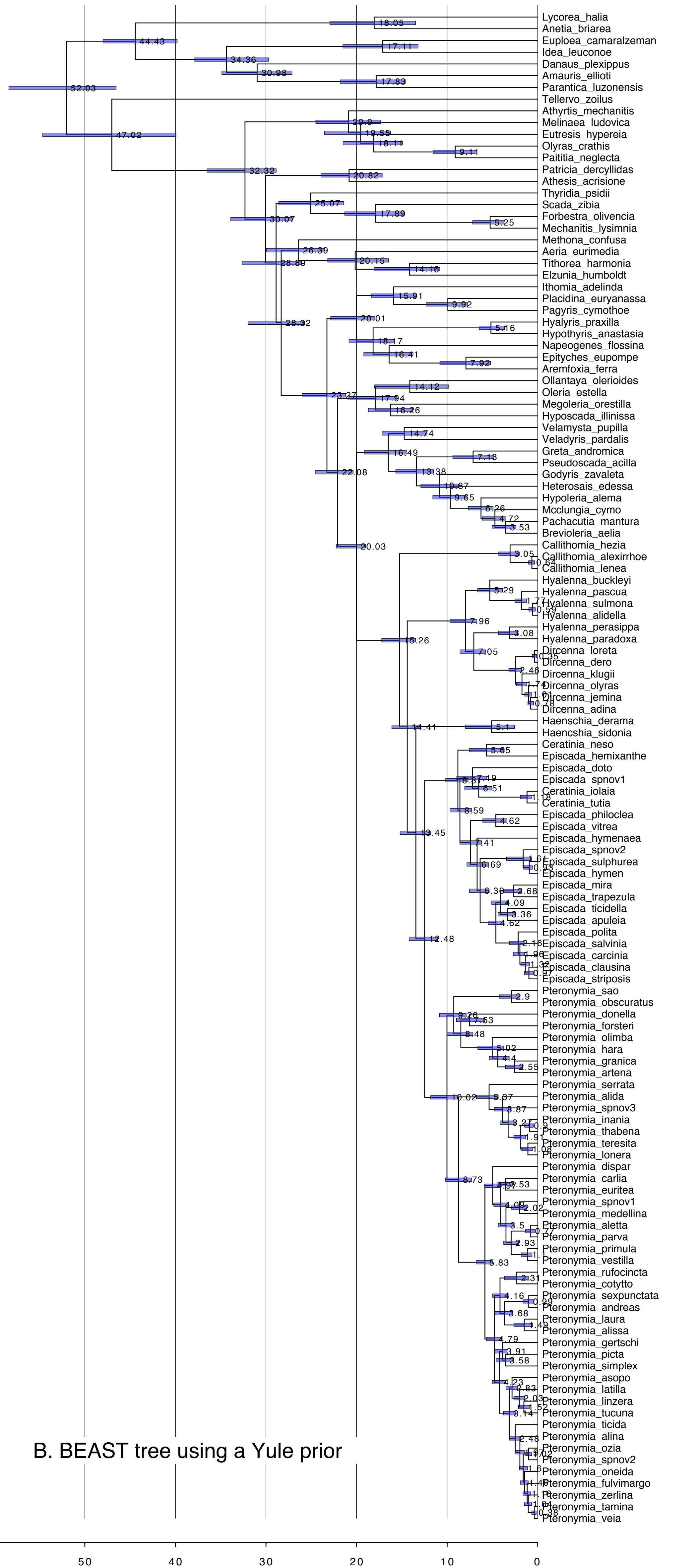

Supplement: Supplementary file 4 [file ECE3-8-3965-s004.pdf]
