# Supplementary material for: Contrasting patterns of Andean diversification among three diverse clades of Neotropical clearwing butterflies
Source: Ecol Evol. 2018 Mar 25;8(8):3965–82. doi: 10.1002/ece3.3622 (PMC5916281; doi:10.1002/ece3.3622)
Supplement: Supplementary file 5 [file ECE3-8-3965-s005.pdf]

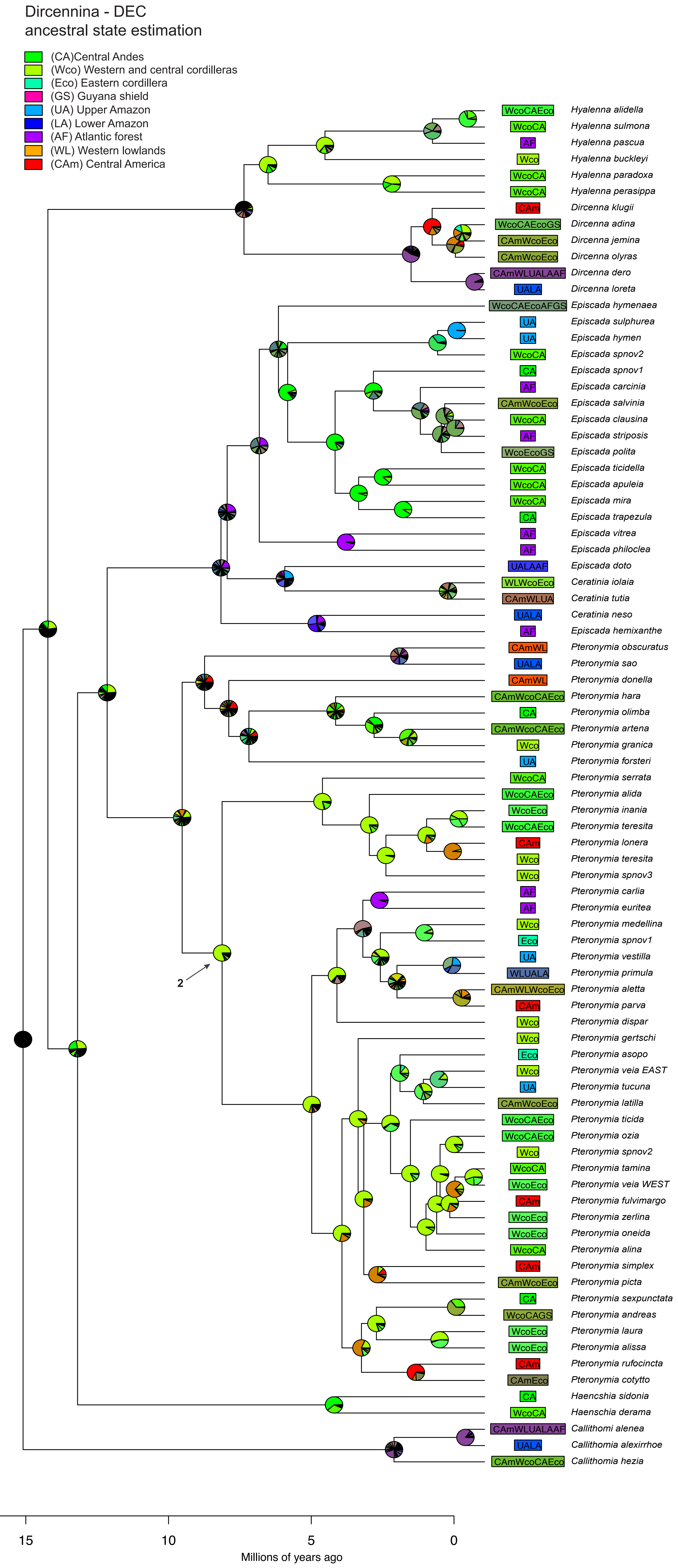

Dircennina - DEC
ancestral state estimation
(CA)Central Andes
(Wco) Western and central cordilleras
(Eco) Eastern cordillera
(GS) Guyana shield
(UA) Upper Amazon
(LA) Lower Amazon
(AF) Atlantic forest
(WL) Western lowlands
(CAm) Central America
WcoCAEco Hyalenna alidella
WcoCA Hyalenna sulmona
AF Hyalenna pascua
Wco Hyalenna buckleyi
WcoCA Hyalenna paradoxa
WcoCA Hyalenna perasippa
CAm Dircenna klugii
WcoCAEcoGS Dircenna adina
CAmWcoEco Dircenna jemina
CAmWcoEco Dircenna olyras
CAmWLUALAAF Dircenna dero
UALA Dircenna loreta
WcoCAEcoAFGS Episcada hymenaea
UA Episcada sulphurea
UA Episcada hymen
WcoCA Episcada spnov2
CA Episcada spnov1
AF Episcada carcinia
CAmWcoEco Episcada salvinia
WcoCA Episcada clausina
AF Episcada striposis
WcoEcoGS Episcada polita
WcoCA Episcada ticidella
WcoCA Episcada apuleia
WcoCA Episcada mira
CA Episcada trapezula
AF Episcada vitrea
AF Episcada philoclea
UALAAF Episcada doto
WLWcoEco Ceratinia iolaia
CAmWLUA Ceratinia tutia
UALA Ceratinia neso
AF Episcada hemixanthe
CAmWL Pteronymia obscuratus
UALA Pteronymia sao
CAmWL Pteronymia donella
CAmWcoCAEco Pteronymia hara
CA Pteronymia olimba
CAmWcoCAEco Pteronymia artena
Wco Pteronymia granica
UA Pteronymia forsteri
WcoCA Pteronymia serrata
WcoCAEco Pteronymia alida
WcoEco Pteronymia inania
WcoCAEco Pteronymia teresita
CAm Pteronymia lonera
Wco Pteronymia teresita
Wco Pteronymia spnov3
AF Pteronymia carlia
AF Pteronymia euritea
Wco Pteronymia medellina
Eco Pteronymia spnov1
UA Pteronymia vestilla
WLUALA Pteronymia primula
CAmWLWcoEco Pteronymia aletta
CAm Pteronymia parva
Wco Pteronymia dispar
Wco Pteronymia gertschi
Eco Pteronymia asopo
Wco Pteronymia veia EAST
UA Pteronymia tucuna
CAmWcoEco Pteronymia latilla
WcoCAEco Pteronymia ticida
WcoCAEco Pteronymia ozia
Wco Pteronymia spnov2
WcoCA Pteronymia tamina
WcoEco Pteronymia veia WEST
CAm Pteronymia fulvimargo
WcoEco Pteronymia zerlina
WcoEco Pteronymia oneida
WcoCA Pteronymia alina
CAm Pteronymia simplex
CAmWcoEco Pteronymia picta
CA Pteronymia sexpunctata
WcoCAGS Pteronymia andreas
WcoEco Pteronymia laura
WcoEco Pteronymia alissa
CAm Pteronymia rufocincta
CAmEco Pteronymia cotytto
CA Haencshia sidonia
WcoCA Haenschia derama
CAmWLUALAAF Callithomi alenea
UALA Callithomia alexirrhoe
CAmWcoCAEco Callithomi hezia
2
15
10
5
0
Millions of years ago

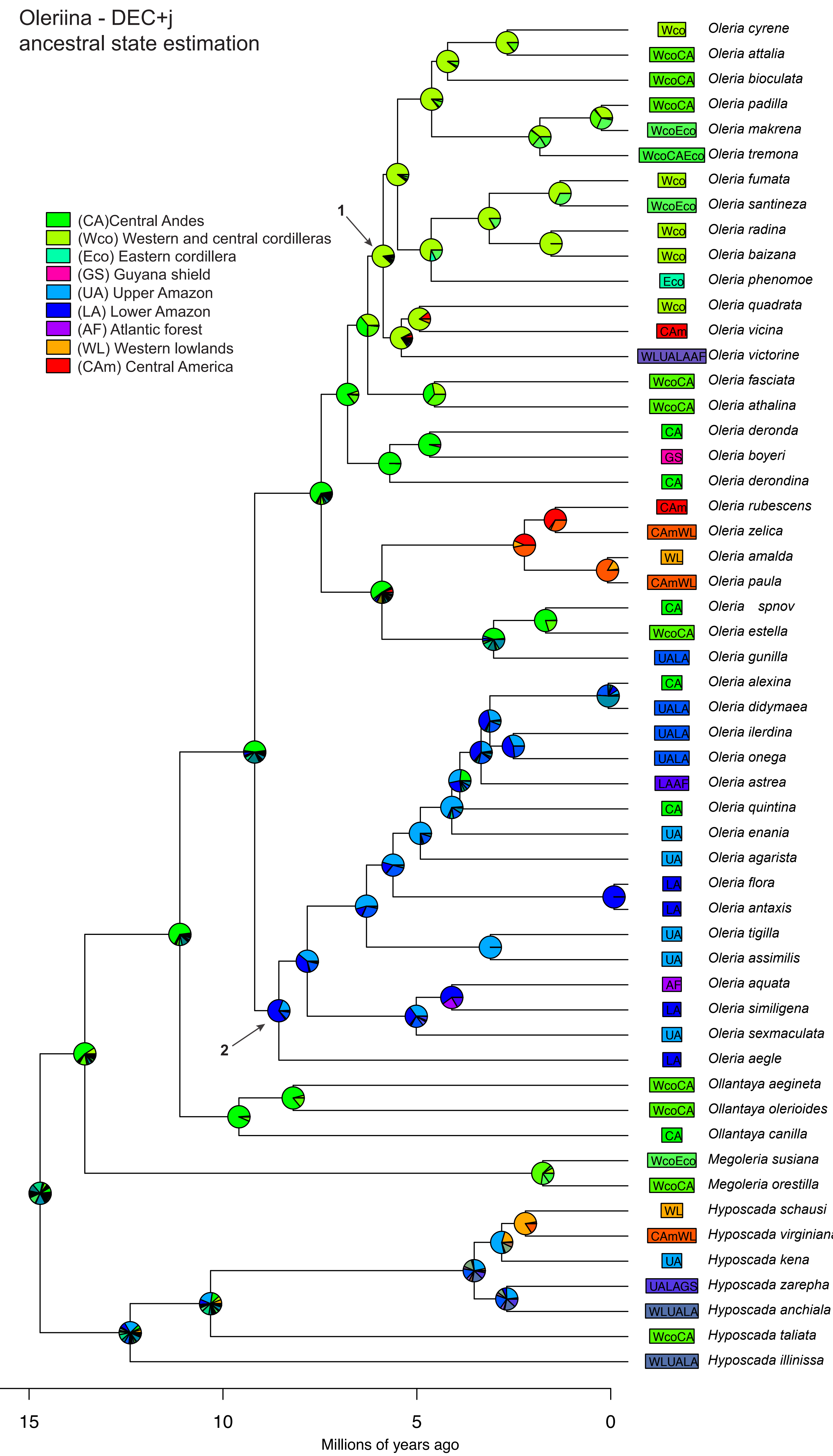
Oleriina - DEC+j
ancestral state estimation
(CA)Central Andes
(Wco) Western and central cordilleras
(Eco) Eastern cordillera
(GS) Guyana shield
(UA) Upper Amazon
(LA) Lower Amazon
(AF) Atlantic forest
(WL) Western lowlands
(CAm) Central America
1
2
Wco Oleria cyrene
WcoCA Oleria attalia
WcoCA Oleria bioculata
WcoCA Oleria padilla
WcoEco Oleria makrena
WcoCAEco Oleria tremona
Wco Oleria fumata
WcoEco Oleria santineza
Wco Oleria radina
Wco Oleria baizana
Eco Oleria phenomoe
Wco Oleria quadrata
CAm Oleria vicina
WLUALAAF Oleria victorine
WcoCA Oleria fasciata
WcoCA Oleria athalina
CA Oleria deronda
GS Oleria boyeri
CA Oleria derondina
CAm Oleria rubescens
CAmWL Oleria zelica
WL Oleria amalda
CAmWL Oleria paula
CA Oleria spnov
WcoCA Oleria estella
UALA Oleria gunilla
CA Oleria alexina
UALA Oleria didymaea
UALA Oleria ilerdina
UALA Oleria onega
LAAF Oleria astrea
CA Oleria quintina
UA Oleria enania
UA Oleria agarista
LA Oleria flora
LA Oleria antaxis
UA Oleria tigilla
UA Oleria assimilis
AF Oleria aquata
LA Oleria similigena
UA Oleria sexmaculata
LA Oleria aegle
WcoCA Ollantaya aegineta
WcoCA Ollantaya olerioides
CA Ollantaya canilla
WcoEco Megoleria susiana
WcoCA Megoleria orestilla
WL Hyposcada schausi
CAmWL Hyposcada virginiana
UA Hyposcada kena
UALAGS Hyposcada zarepha
WLUALA Hyposcada anchiala
WcoCA Hyposcada taliata
WLUALA Hyposcada illinissa
15
10
5
0
Millions of years ago
