# Supplementary material for: Contrasting patterns of Andean diversification among three diverse clades of Neotropical clearwing butterflies
Source: Ecol Evol. 2018 Mar 25;8(8):3965–82. doi: 10.1002/ece3.3622 (PMC5916281; doi:10.1002/ece3.3622)
Supplement: Supplementary file 6 [file ECE3-8-3965-s006.docx]

Supporting Information S1. (Separate file) List of all individuals, species and Genbank accession codes included in this study.

Supporting Information S2. (Separate file) IQ-TREE including all individuals and outgroups. Numbers in parentheses are SH-aLRT support (%) / ultrafast bootstrap support (%).

Supporting Information S3. Secondary calibrations used to time-calibrate the phylogeny of the Dircennina. We used uniform prior bounded by the *Calibration interval*.

| Node calibrated | Calibration interval | Hostplant | Source |
| --- | --- | --- | --- |
| MRCA *Danaus plexippus – Amauris Ellioti* | 35 - 24.5 mya | NA | Wahlberg et al. 2009 |
| Crown Athesitina | 24 – 0 mya | *Capsicum* | De Silva et al. (2017) |
| Crown Danainae | 60 – 44 mya | NA | Wahlberg et al. 2009 |
| Crown Danaini | 48 -35 mya | NA | Wahlberg et al. 2009 |
| MRCA Dircennina - Godyridina | 24 – 19 mya | NA | Wahlberg et al. 2009 |
| Crown Ithomiini | 83 – 0 mya | Solanaceae | Magallon et al. (2015) |
| Crown Melinaeina | 24 – 0 mya | *Schultesianthus* | De Silva et al. (2017) |
| MRCA *Pagyris* - *Placidina* | 14 – 0 mya | *Brugmansia* | De Silva et al. (2017) |

Supporting Information S4. (Separate file) Dircennina species time-tree calibrated with Beast v1.8.2, including outgroups. Bayesian posterior probabilities are indicated at the nodes.

Supporting Information S5. (Separate file) Dircennina species time-tree calibrated with Beast v1.8.2 including outgroups. Median node ages are indicated at nodes as well as the 95% height posterior density. A. BEAST tree using a Birth-Death prior (tree used in all analyses). B. BEAST tree using a Yule prior (for comparison).

Supporting Information S6. Biogeographic model implemented in BiogeoBEARS and ancestral state estimation represented with pie-charts.

CAm: Central America

WL: Western Andean lowlands/Magdalena valley

Wco: Northern Andes, Western and Central cordilleras

CA: Central Andes

Eco: Northern Andes, Eastern cordilleras

UA: Upper-Amazon

LA: Lower-Amazon

AF: Atlantic-Forest

GS: Guiana Shield

1. Combinations of areas allowed

|  | CAm | WL | Wco | CA | Eco | UA | LA | AF | GS |
| --- | --- | --- | --- | --- | --- | --- | --- | --- | --- |
| CAm | 1 | 1 | 1 | 1 | 1 | 1 | 1 | 1 | 1 |
| WL | 1 | 1 | 1 | 1 | 1 | 1 | 1 | 1 | 0 |
| Wco | 1 | 1 | 1 | 1 | 1 | 1 | 0 | 1 | 1 |
| CA | 1 | 1 | 1 | 1 | 1 | 1 | 0 | 1 | 0 |
| Eco | 1 | 1 | 1 | 1 | 1 | 1 | 1 | 0 | 1 |
| UA | 1 | 1 | 1 | 1 | 1 | 1 | 1 | 1 | 1 |
| LA | 1 | 1 | 0 | 0 | 1 | 1 | 1 | 1 | 1 |
| AF | 1 | 1 | 1 | 1 | 0 | 1 | 1 | 1 | 1 |
| GS | 1 | 0 | 1 | 0 | 1 | 1 | 1 | 1 | 1 |

1. (Separate file) Results of ancestral state estimation represented with pie-charts for Dircennina and Oleriina.

Supporting Information S7. BISSE ancestral state reconstruction of Oleriina.

ClaSSE models cannot be used for ancestral state reconstruction. Thus we used the BiSSE model, which can be used for ancestral state reconstruction, but only allows character state changes (biogeographic region in our case) to occur on branches. We fitted BiSSE models equivalent to the four best ClaSSE models. For the “null” model of Oleriina, parameter estimates of BiSSE were congruent with ClaSSE and resulted in an ancestral state reconstruction similar to that of BiogeoBEARS (Figure 2. in the manuscript, Figure S7.B). When fitting BiSSE parameters corresponding to the three other best fitting ClaSSE models, the trend inferred for colonization parameters were opposite, i.e. higher colonization rates out of the Andes than into the Andes (Table S7). As a result, the ancestral state reconstructions using BiSSE parameter estimates (hereafter referred to as “unconstrained”) inferred an Andean origin followed by multiple colonization of non-Andean regions (Figure S7.A C D), a pattern similar to that identified with the null model and BiogeoBEARS (Figure 1 in the manuscript). We tried to constrain the BiSSE models with parameter values estimated with ClaSSE (hereafter referred to as “constrained”) and performed the ancestral state reconstruction (Figure S7.A C D.). However this ancestral state reconstruction largely differed from the null BiSSE model and BiogeoBEARS, mostly as a consequence of higher colonization rate toward the Andes than out of the Andes.

Maximum likelihood analyses indicated that these ClaSSE models with speciation, colonization or extinction parameters free to vary were not significantly different than the “null” model (Table 1 in the article), which had the lowest number of parameters (only three). MCMC analyses showed than the differences among these parameters are very small when they are allowed to vary (Figure 4). Besides, the absence of congruence with the BiogeBEARS reconstruction suggests that the parameters estimated by the ClaSSE models were unreliable. Therefore we chose to interpret in the manuscript the “null” model, which has a similar explanatory power with fewer parameters and is congruent with the BiogeoBEARS ancestral state reconstruction.

Table S7. Results when BiSSE parameters were fitted for the models having equivalent explanatory power than the null model in the manuscript. Note that colonization rate (q12 and q21) have opposite results from ClaSSE, i.e higher colonization rates out of the Andes than into the Andes. λ1=speciation rate in non-Andean regions, λ2=speciation rate within the Andes, µ1/ µ2=extinction rate, q12=colonization rate into the Andes, q21=colonization rate out of the Andes.

| **parameters free to vary** | **λ1** | **λ2** | **µ1** | **µ2** | **q12** | **q21** |
| --- | --- | --- | --- | --- | --- | --- |
| colonization | 1.93e-01 |  | 2.40e-08 |  | 2.92e-02 | 5.45e-02 |
| null (λ ≠ q) | 1.93e-01 |  | 4.63 e-08 |  | 4.89e-02 |  |
| speciation and colonization | 1.95e-01 | 1.92e-01 | 5.786e-08 |  | 2.917e-02 | 5.442e-02 |
| colonization and extinction | 1.93e-01 |  | 1.51e-06 | 3.06e-07 | 2.92e-02 | 5.45e-02 |

Figure S7. A. Ancestral state reconstructions in Oleriina with BiSSE unconstrained (left) and constrained (right). Colonization parameters are free to vary. Blue=non-Andean. Red=Andean.

Figure S7. B. Ancestral state reconstructions in Oleriina with BiSSE unconstrained (left) and constrained (right). “Null” model in which speciation is different than colonization parameter. Blue=non-Andean. Red=Andean.

Figure S7. C. Ancestral state in Oleriina reconstructions with BiSSE unconstrained (left) and constrained (right). Colonization and speciation parameters are free to vary. Blue=non-Andean. Red=Andean.

Figure S7. D. Ancestral state reconstruction in Olerina with BiSSE unconstrained (left) and constrained (right). Colonization and extinction parameters are free to vary. Blue=non-Andean. Red=Andean.

Supporting Information S8. Results of all the time-dependent models of diversification fitted on the different partitions: 0 shift, 1 shift, 2 shifts. P(LRT) is the p value of the likelihood ratio test compared to the null model. BCST=constant speciation, BVAR=time-dependent speciation, DCST=constant extinction, DVAR time-dependent extinction. λ =speciation rate at present, α=coefficient of time variation of the speciation rate, µ =extinction rate at present, ß =coefficient of time variation of the extinction rate. Bold indicates the models used and discussed in the manuscript.

1- Dircennina

1- a. Whole tree

| Model | Parameters | logL | AIC | P(LRT) | λ | α | µ | ß |
| --- | --- | --- | --- | --- | --- | --- | --- | --- |
| **BCST** | **1** | **-198.53** | **399.07** | **nullmodel** | **0.264** |  |  |  |
| BVAR | 2 | -198.45 | 400.91 | 0.688 | 0.252 | 0.0130 |  |  |
| BCSTDCST | 2 | -198.53 | 401.07 | 1 | 0.264 |  | 2.60E-07 |  |
| BVARDCST | 3 | -198.45 | 402.91 | 0.922 | 0.252 | 0.0130 | 3.37E-07 |  |
| BCSTDVAR | 3 | -198.53 | 403.07 | 1 | 0.264 |  | 1.59E-07 | 0.0019 |
| BVARDVAR | 4 | -198.45 | 404.91 | 0.983 | 0.252 | 0.0131 | 2.07E-07 | 0.0715 |

1- b. Background tree excluding *Pteronymia*-group

| Model | Parameters | logL | AIC | P(LRT) | λ | α | µ | ß |
| --- | --- | --- | --- | --- | --- | --- | --- | --- |
| BVARDVAR | 4 | -131.45 | 270.91 | 0.078 | 0.1597 | 0.4007 | 0.1848 | 0.3897 |
| **BCST** | **1** | **-134.85** | **271.70** | **nullmodel** | **0.2257** |  |  |  |
| BVAR | 2 | -134.65 | 273.30 | 0.524 | 0.2035 | 0.0240 |  |  |
| BCSTDCST | 2 | -134.85 | 273.70 | 1 | 0.2257 |  | 2.51E-07 |  |
| BVARDCST | 3 | -134.63 | 275.26 | 0.802 | 0.2121 | 0.0326 | 0.0358 |  |
| BCSTDVAR | 3 | -134.85 | 275.70 | 1 | 0.2260 |  | 1.04E-06 | 0.0134 |

1- c. *Pteronymia*-group

| Model | Parameters | logL | AIC | P(LRT) | λ | α | µ | ß |
| --- | --- | --- | --- | --- | --- | --- | --- | --- |
| BVAR | 2 | -59.24 | 122.49 | 0.071 | 0.2616 | 0.1733 |  |  |
| **BCST** | **1** | **-60.87** | **123.74** | **nullmodel** | **0.3843** |  |  |  |
| BVARDCST | 3 | -59.24 | 124.49 | 0.196 | 0.2617 | 0.1734 | 1.04E-07 |  |
| BCSTDCST | 2 | -60.87 | 125.74 | 1 | 0.3845 |  | 7.85E-08 |  |
| BVARDVAR | 4 | -59.24 | 126.49 | 0.354 | 0.2617 | 0.1734 | 1.04E-07 | 0.01 |
| BCSTDVAR | 3 | -60.87 | 127.74 | 1 | 0.3843 |  | 1.04E-07 | 0.01 |

2- Oleriina

2- a. Whole tree

| Model | Parameters | logL | AIC | P(LRT) | λ | α | µ | ß |
| --- | --- | --- | --- | --- | --- | --- | --- | --- |
| **BCST** | **1** | **-140.43** | **282.87** | **nullmodel** | **0.188** |  |  |  |
| BVAR | 2 | -139.47 | 282.94 | 0.178 | 0.129 | 0.086 |  |  |
| BCSTDCST | 2 | -140.43 | 284.87 | 1 | 0.188 |  | 2.20E-08 |  |
| BVARDCST | 3 | -139.47 | 284.94 | 0.405 | 0.129 | 0.086 | 3.95E-08 |  |
| BCSTDVAR | 3 | -140.43 | 286.87 | 1 | 0.188 |  | 5.21E-08 | -0.030 |
| BVARDVAR | 4 | -139.47 | 286.94 | 0.613 | 0.129 | 0.086 | 2.11E-07 | 0.020 |

2- b. Background tree excluding the onega-group

| Model | Parameters | logL | AIC | P(LRT) | λ | α | µ | ß |
| --- | --- | --- | --- | --- | --- | --- | --- | --- |
| **BCST** | **1** | **-100.71** | **203.42** | **nullmodel** | **0.181** |  |  |  |
| BCSTDCST | 2 | -100.71 | 205.42 | 1 | 0.181 |  | 4.67E-08 |  |
| BVAR | 2 | -101.03 | 206.06 | 1 | 0.132 | 0.071 |  |  |
| BCSTDVAR | 3 | -100.71 | 207.42 | 1 | 0.181 |  | 1.71E-07 | -0.022 |
| BVARDCST | 3 | -101.03 | 208.06 | 1 | 0.132 | 0.070 | 3.04E-08 |  |
| BVARDVAR | 4 | -101.03 | 210.06 | 1 | 0.132 | 0.070 | 1.85E-08 | -0.015 |

2- c. Onega-group

| Model | Parameters | logL | AIC | P(LRT) | λ | α | µ | ß |
| --- | --- | --- | --- | --- | --- | --- | --- | --- |
| **BVAR** | **2** | **-36.92** | **77.84** | **0.020** | **0.0821** | **0.2392** |  |  |
| BVARDCST | 3 | -36.92 | 79.84 | 0.067 | 0.0821 | 0.2392 | 1.14E-07 |  |
| BCST | 1 | -39.61 | 81.22 | nullmodel | 0.2053 |  |  |  |
| BVARDVAR | 4 | -36.92 | 81.84 | 0.146 | 0.0821 | 0.2392 | 3.42E-08 | 0.0134 |
| BCSTDCST | 2 | -39.61 | 83.22 | 1 | 0.2053 |  | 3.47E-08 |  |
| BCSTDVAR | 3 | -39.61 | 85.22 | 1 | 0.2053 |  | 3.42E-08 | 0.0134 |

2- d. Background tree excluding the makrena-group

| Model | Parameters | logL | AIC | P(LRT) | λ | α | µ | ß |
| --- | --- | --- | --- | --- | --- | --- | --- | --- |
| **BCST** | **1** | **-107.52** | **217.04** | **nullmodel** | **0.172** |  |  |  |
| BVAR | 2 | -107.26 | 218.51 | 0.469 | 0.116 | 0.084 |  |  |
| BCSTDCST | 2 | -107.52 | 219.04 | 1 | 0.172 |  | 8.06E-07 |  |
| BVARDCST | 3 | -107.26 | 220.51 | 0.769 | 0.117 | 0.084 | 3.02E-07 |  |
| BCSTDVAR | 3 | -107.52 | 221.04 | 1 | 0.172 |  | 6.43E-08 | -0.028 |
| BVARDVAR | 4 | -107.26 | 222.51 | 0.9133 | 0.116 | 0.084 | 1.94E-07 | 0.000 |

2- e. Makrena-group

| Model | Parameters | logL | AIC | P(LRT) | λ | α | µ | ß |
| --- | --- | --- | --- | --- | --- | --- | --- | --- |
| **BVAR** | **2** | **-27.68** | **59.35** | **0.003** | **0.053** | **0.481** |  |  |
| BVARDCST | 3 | -27.37 | 60.75 | 0.009 | 0.092 | 0.505 | 0.369 |  |
| BCSTDVAR | 3 | -28.41 | 62.83 | 0.025 | 0.727 |  | 2090.652 | -745.091 |
| BVARDVAR | 4 | -28.19 | 64.39 | 0.050 | 0.559 | 0.089 | 2091.485 | -745.133 |
| BCST | 1 | -32.10 | 66.21 | nullmodel | 0.253 |  |  |  |
| BCSTDCST | 2 | -32.10 | 68.21 | 0.999 | 0.252 |  | 4.08E-08 |  |

2- f. Background group excluding the onega-group and the makrena-group

| Model | Parameters | logL | AIC | P(LRT) | λ | α | µ | ß |
| --- | --- | --- | --- | --- | --- | --- | --- | --- |
| **BCST** | **1** | **-67.51** | **137.02** | **nullmodel** | **0.155** |  |  |  |
| BVAR | 2 | -66.72 | 137.44 | 0.208 | 0.109 | 0.065 |  |  |
| BVARDVAR | 4 | -64.87 | 137.74 | 0.153 | 0.080 | 0.370 | 0.108 | 0.347 |
| BCSTDCST | 2 | -67.51 | 139.02 | 1.000 | 0.155 |  | 0.000 |  |
| BVARDCST | 3 | -66.67 | 139.34 | 0.432 | 0.121 | 0.081 | 0.065 |  |
| BCSTDVAR | 3 | -67.51 | 141.02 | 1.000 | 0.155 |  | 1.90E-08 | 0.0001 |
